# Attenuated humoral responses in HIV after SARS-CoV-2 vaccination linked to B cell defects and altered immune profiles

**DOI:** 10.1016/j.isci.2022.105862

**Published:** 2022-12-24

**Authors:** Emma Touizer, Aljawharah Alrubayyi, Rosemarie Ford, Noshin Hussain, Pehuén Pereyra Gerber, Hiu-Long Shum, Chloe Rees-Spear, Luke Muir, Ester Gea-Mallorquí, Jakub Kopycinski, Dylan Jankovic, Anna Jeffery-Smith, Christopher L. Pinder, Thomas A. Fox, Ian Williams, Claire Mullender, Irfaan Maan, Laura Waters, Margaret Johnson, Sara Madge, Michael Youle, Tristan J. Barber, Fiona Burns, Sabine Kinloch, Sarah Rowland-Jones, Richard Gilson, Nicholas J. Matheson, Emma Morris, Dimitra Peppa, Laura E. McCoy

**Affiliations:** 1 Institute for Immunity and Transplantation, Division of Infection and Immunity, University College London, London, UK; 2 Nuffield Department of Medicine, University of Oxford, Oxford, UK; 3 Cambridge Institute of Therapeutic Immunology and Infectious Disease, Department of Medicine, University of Cambridge, Cambridge, UK; 4 Mortimer Market Centre, Department of HIV, Central and North West London NHS Trust, London, UK; 5 Institute for Global Health, University College London, London, UK; 6 The Ian Charleson Day Centre, Royal Free Hospital NHS Foundation Trust, London, UK; 7 NHS Blood and Transplant, Cambridge, UK

**Keywords:** Immunology, Virology

## Abstract

We assessed a cohort of people living with human immunodeficiency virus (PLWH) (n = 110) and HIV negative controls (n = 64) after 1, 2 or 3 SARS-CoV-2 vaccine doses. At all timepoints, PLWH had significantly lower neutralizing antibody (nAb) titers than HIV-negative controls. We also observed a delayed development of neutralization in PLWH that was underpinned by a reduced frequency of spike-specific memory B cells (MBCs). Improved neutralization breadth was seen against the Omicron variant (BA.1) after the third vaccine dose in PLWH but lower nAb responses persisted and were associated with global MBC dysfunction. In contrast, SARS-CoV-2 vaccination induced robust T cell responses that cross-recognized variants in PLWH. Strikingly, individuals with low or absent neutralization had detectable functional T cell responses. These PLWH had reduced numbers of circulating T follicular helper cells and an enriched population of CXCR3^+^CD127^+^CD8^+^T cells after two doses of SARS-CoV-2 vaccination.

## Highlights

• PLWH have lower levels of neutralizing antibodies (nAbs) after SARS-CoV-2 vaccination
• Delayed production of nAbs is associated with greater memory B cell (MBCs) disturbance
• Additional doses increase nAbs titers and breadth despite persistent MBCs perturbations
• SARS-CoV-2 vaccination elicits robust T cell responses in PLWH
• PLWH with spike-specific T cells but no nAbs have increased CXCR3+CD127+CD8^+^T cells

## Graphical abstract

**Figure:**
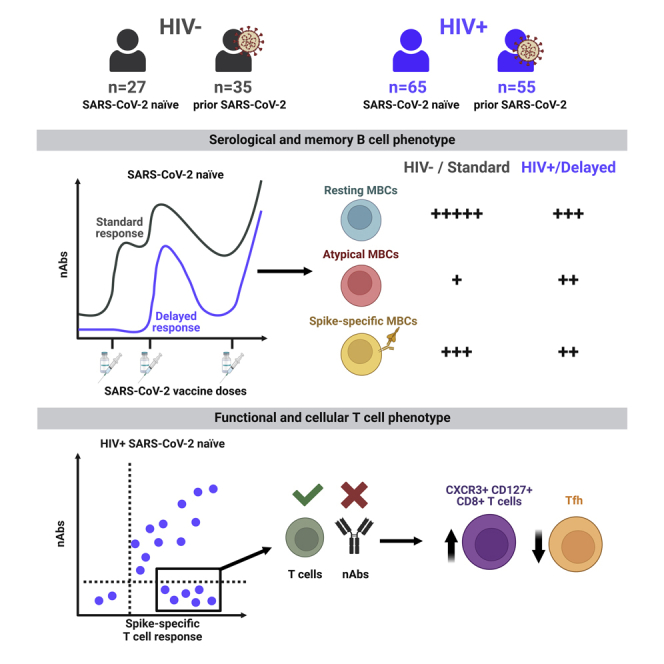

## Introduction

People living with human immunodeficiency virus (HIV)[PLWH] appear to be at a higher risk of hospitalization and worse clinical outcomes from COVID-19 disease, especially in the context of cellular immunosuppression and unsuppressed HIV viral load.^1^ Although combined antiretroviral therapy (cART) has dramatically improved life expectancy in PLWH, the persistence of immune dysfunction raises concerns about the overall effectiveness and durability of vaccine responses in this potentially more vulnerable patient group, in line with other immunocompromised groups^2^^,^^3^ As a result, PLWH were included in either priority group 4 (for clinically vulnerable PLWH, based on more advanced immunosuppression or co-morbidities) or 6 (all other PLWH)^4^ in the UK for earlier COVID-19 vaccination than the general population. The Joint Committee on Vaccination and Immunization (JCVI) advised to invite this patient group for an additional booster dose.^4^ Previously, defects have been observed in serological vaccine responses in PLWH on cART. For example, after a full course of hepatitis B^5^ or influenza vaccination^6^ and long-term responses to vaccination can be shorter-lived in PLWH compared to the general population.^7^ We and others have previously shown a failure to mount a robust antibody response following COVID-19 vaccination in advanced HIV infection with low CD4 T cell counts below 200 cells/μL.^8^^,^^9^^,^^10^^,^^11^^,^^12^

Data on vaccine efficacy and immunogenicity in PLWH remains limited (reviewed in^13^), and although there are some conflicting results, meta-analyses^14^ and recent studies^15^ have shown reduced levels of seroconversion and neutralization after a second dose of viral vector vaccine dose in PLWH receiving cART, with lower CD4 T cell count/viremia and older age resulting in a more impaired response and more rapid breakthrough infection.^16^ Assessment of vaccine efficacy has been continually complicated by the ongoing emergence of variants of concern (VOC), with the Alpha, Beta, Delta and Omicron variants being observed to progressively evade antibodies^17^^,^^18^ raised against the original Wuhan-Hu-1 strain in most vaccines. In particular, data after three vaccine doses has been hard to assess because of the emergence of Omicron concurrent with vaccination, and in PLWH data on humoral and cellular responses including against Omicron remains limited.^19^^,^^20^^,^^21^^,^^22^ However, the data available thus far suggest that the third vaccine dose provides a strong boost to antibody responses regardless of the CD4 T cell count, including in those who had previously not seroconverted and induces robust T cell responses.^19^^,^^21^^,^^22^ Moreover, most studies on SARS-CoV-2 vaccine responses in PLWH to date have mostly focused on evaluating humoral responses and generated limited data on functional T cell responses after 2 doses^23^ or 3 doses.^19^^,^^20^ Therefore, it remains unclear what role HIV-associated immune dysfunction plays in serological and cellular outcome after SARS-CoV-2 vaccination.

Inferior serological responses to vaccination in PLWH (on cART or untreated) are most commonly linked to HIV-induced immune destruction of CD4 T cells and imbalance of the CD4:CD8 T cell populations.^24^^,^^25^ Despite effective cART, chronic immune activation in HIV can lead to exhaustion of the adaptive immune system.^26^ This can translate into impaired T cell responses, likely limiting T follicular helper (T_FH_) cell help to B cells, resulting in lower serological outputs. There is also substantial evidence for dysfunction/exhaustion in the B cell compartment during chronic infections that may limit antibody responses against the infecting pathogen.^27^^,^^28^ This B cell dysfunction persists to a variable degree after HIV viral suppression following cART initiation,^29^^,^^30^ but how these B cell defects impact serological responses to vaccination has not yet been fully elucidated. Furthermore, there is substantial age-related decline in immune function leading to senescence in both the T and B cell compartments, which may be accelerated in PLWH and could further influence vaccine responses.^31^

In this study we have evaluated in a well-curated cohort of PLWH on cART and HIV-negative controls following three SARS-CoV-2 vaccine doses, the relationship between humoral and functional T cell responses. To achieve this goal, we have assessed how spike-specific memory B cell (MBC) responses, global MBC profiles, CD4 and CD8 T cell phenotypes are linked with serological outcomes in PLWH to better understand which factors may modulate immune responses to vaccination.

## Results

### Lower levels of seroconversion and neutralizing antibodies after SARS-CoV-2 immunization in PLWH without a history of prior COVID-19 disease

**Figure 1:**
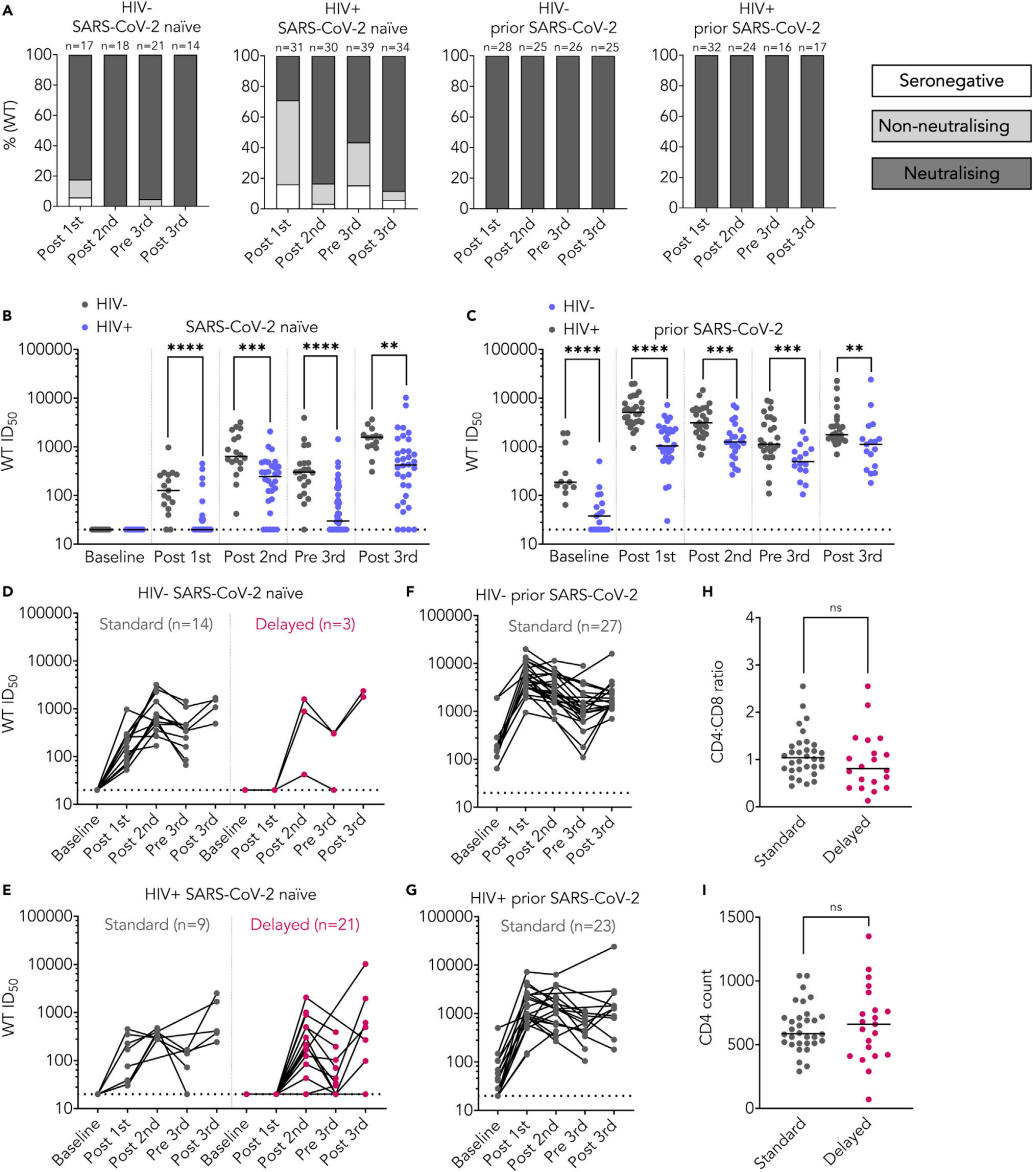
Weaker post vaccination antibody responses in SARS-CoV-2 naive PLWH (A) Percentage of individuals with detectable neutralizing antibody response, non-neutralizing but binding response, or seronegative at each timepoint as color-coded in the key. The headings above each graph show HIV status and previous SARS-CoV-2 exposure. N numbers for each group are indicated above each column. (B) WT pseudovirus neutralization reciprocal 50% inhibitory titers (ID50) in PLWH (blue) compared to HIV-negative controls (gray) stratified by vaccination timepoint (on the x axis) for individuals without prior SARS-CoV-2 infection. The dotted line represents the lower limit of the assay (ID50 = 1:20). Where no neutralization was detected, samples were assigned an ID50 of <1:20 as this was the limit of assay detection. Each data point represents the mean of n = 2 biological repeats, each measured in duplicates. N numbers match those in (A). Line represents median for each group. Statistical test: Mann Whitney U-test (MWU). (C) Shows the equivalent data for those with prior SARS-CoV-2 infection, N numbers match those in (A). (D) Longitudinal ID_50_ titers for HIV-negative controls without prior SARS-CoV-2 infection who provided at least two longitudinal samples, including a post first dose sample. Samples that were neutralizing after the first dose are categorized as exhibiting a standard neutralizing response and colored gray, those that only achieve neutralization after the second dose, exhibit a delayed neutralizing response and are color-coded in magenta. N numbers for each category are indicated on the graph. (E) Shows the equivalent data for PLWH without prior SARS-CoV-2 infection. (F) Shows the equivalent data for HIV-negative controls with prior SARS-CoV-2 infection. (G) Shows the equivalent data for PLWH with prior SARS-CoV-2 infection. (H) CD4 T cell counts and (I) CD4:CD8 T cell ratio for PLWH stratified by standard (gray) or delayed neutralization (magenta). N numbers are as per D-G. Line represents median for each group. Statistical test: MWU. ^∗^p > 0.05; ^∗^^∗^p > 0.01; ^∗^^∗∗^p > 0.001 and ^∗∗^^∗∗^p > 0.0001. See also Figure S1.

Participants were recruited between January 2021 and April 2022 (n = 110 PLWH on cART and n = 64 HIV-negative controls) as described in Table 1. Participants were sampled after 1, 2 or 3 doses of a SARS-CoV-2 vaccine and compared cross-sectionally. In addition, in 53 PLWH and 44 controls, responses were assessed longitudinally where sequential samples were available. Both study groups (HIV-negative and PLWH) were divided according to their history of either previous SARS-CoV-2 infection (including infection prior or after vaccination) or as SARS-CoV-2 naive. SARS-CoV-2 spike-specific IgG were tested for binding against the S1 subunit of the SARS-CoV-2 spike protein in a semi-quantitative ELISA^32^^,^^33^ to determine seropositivity. Neutralizing antibodies (nAbs) were measured against the ancestral vaccine-matched Wuhan Hu-1 SARS-CoV-2 (WT) strain by pseudovirus neutralization.^33^ Approximately 90% of HIV-negative controls and 80% of PLWH with no prior history of SARS-CoV-2 infection seroconverted. However, although over 82% of controls produced a neutralizing response after one vaccine dose, only 29% of PLWH did so (Figure 1A). As described,^34^ prior history of SARS-CoV-2 infection was associated with a higher level of seroconversion and the development of nAbs in all individuals at every studied timepoint regardless of HIV status (Figure 1A).

**Table 1:** Cohort demographics

	HIV- (n = 64)		HIV+ (n = 110)	
	SARS-CoV-2- (n = 27)	SARS-CoV-2+ (n = 37)	SARS-CoV-2- (n = 65)	SARS-CoV-2+ (n = 45)
**Clinical parameters**				

% female	76%	40%	8%	17%
% BAME	28%	24%	21%	44%
Median age (range)	33(21-65)	41(23-66)	53 (22-93)	49 (26-73)
cART	–	–	65 (100%)	45 (100%)
HIV viral load	–	–	Undetectable (<50)	Undetectable (<50)
Median CD4 count (range)	–	–	602 (22-1360)	560(200-1229)
Median CD4:CD8 ratio (range)	–	–	0.74 (0.13–3.05)	0.98 (0.37–2.55)

**Co-morbidities**				

Diabetes, n	–	1	5	3
Hypertension/CVD, n	–	3	3	6
Renal disease, n	–	–	2	1
Liver disease, n	–	–	2	2
Respiratory disease, n	–	1	3	1
Weakened immune system inc. cancer/transplant, n	–	–	8	1
Advanced HIV/HepB co-infection, n	–	–	4	–
Other	–	–	Splenectomy Sarcoidosis	Splenectomy

**Timepoints**				

**Post first dose**				
N =	17	28	31	32
Median days post-previous dose (range)	14 (12-74)	19 (12-60)	20 (12-102)	22 (13-82)
Vaccine (AZ |Moderna |Pfizer)	3 | 2 | 12	3 | 1 | 24	19 | 2 | 10	21 | 0 | 11
**Post second dose**				
N =	18	25	30	24
Median days post-previous dose (range)	39 (23-67)	26 (15-68)	20 (7-48)	21 (9-52)
Vaccine (AZ | Moderna | Pfizer)	3 | 3 | 12	2 | 1 | 23	17 | 1 | 12	12 | 0 | 12
**Pre third dose**				
N =	21	26	39	16
Median days post-previous dose (range)	129 (75-258)	129 (76-236)	125 (72-218)	119 (86-317)
Vaccine (AZ | Moderna | Pfizer)	–	–	–	–
**Post third dose**				
N =	14	25	34	17
Median days post-previous dose (range)	21 (13-43)	43 (18-129)	40 (9-149)	65 (7-140)
Vaccine (AZ |Moderna | Pfizer)	0 | 3 | 12	0 | 2 | 23	1 | 2 | 32	1 | 0 | 16

Cohort demographics, clinical characteristics and number of participants per timepoints for each group. All PLWH participants included in this study were on cART. AZ = AZD1222; Moderna = mRNA-1273; Pfizer = BNT162b2.

Notably, PLWH had lower titers of nAbs than HIV-negative controls at all timepoints regardless of prior SARS-CoV-2 infection (Figures 1B and 1C). Overall, a similar trend was seen in binding responses (Figures S1A and S1B), and nAb titers correlated significantly with both binding titers for S1 IgG and nAb titers obtained from a live virus neutralization assay (Figures S1C and S1D), as previously reported.^35^^,^^36^ Given that this observational cohort includes a mixture of SARS-CoV-2 vaccine types, it was notable that both binding and neutralizing titers remained significantly lower in PLWH compared to controls when only those who had received mRNA-based vaccines were considered (Figures S1E and S1F). A similar analysis for viral vector-based vaccines was not feasible because of insufficient numbers in the control group. Both at the pre- and post-third vaccine dose timepoints, there were more SARS-CoV-2 naive PLWH that fail to produce nAbs (Figures 1A, 1B, and S1A) compared to the control group. Owing to the cross-sectional nature of the analysis, at the pre-third vaccine dose timepoint additional PLWH were recruited. However, the observed differences persisted when PLWH were stratified for co-morbidities (Figure S1G).

Longitudinal samples from 53 PLWH and 44 controls were then evaluated to assess binding antibody responses and nAbs over time after each vaccine dose. These included samples after the first dose and for at least one additional timepoint, often including a baseline, post-second, pre-third and post-third sample (Figure 1D). This analysis revealed two clear trajectories of the development of neutralization, firstly where nAbs were detected after a single vaccine dose,^37^ defined here as “standard neutralization”, and secondly where neutralization was not achieved until after the second dose or later, defined as “delayed neutralization”. Most HIV-negative controls without prior SARS-CoV-2 infection show a standard neutralization profile, with only 3 individuals failing to mount a neutralizing response until after the second dose (Figure 1D), and a similar effect was seen with binding responses (Figures S1H–S1K). In contrast, two-thirds of SARS-CoV-2 naive PLWH did not make a detectable neutralizing response until after the second dose and a substantial proportion of them lost detectable neutralizing activity before the third dose (Figures 1A and 1E). However, both PLWH and HIV-negative controls with a history of SARS-CoV-2 infection made a standard neutralizing response (Figures 1F and 1G). Therefore, having identified this delayed neutralization phenotype in SARS-CoV-2 naive PLWH, we have evaluated its relationship with total CD4 T cell counts, which are known to be important for SARS-CoV-2 vaccine responses in PLWH.^8^^,^^9^^,^^10^^,^^12^ No significant difference was seen in median CD4 T cell count or CD4:CD8 T cell ratio between PLWH with standard or delayed neutralization profiles (Figures 1H and 1I); or correlate either with the rapid development of neutralization (Figure S1M and S1N).

### Delayed neutralization is associated with lower frequency of spike-specific MBCs and a perturbed MBC global phenotype

**Figure 2:**
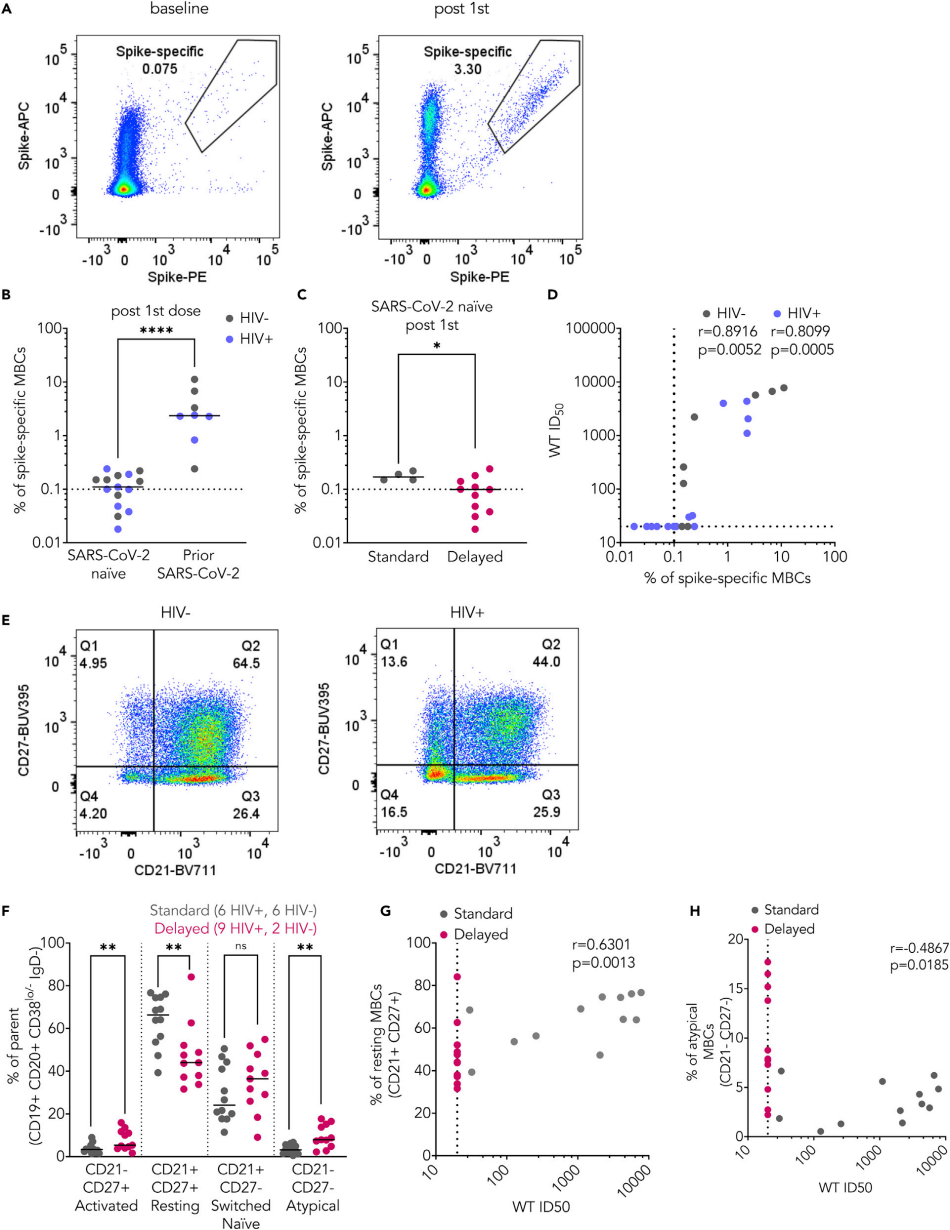
Neutralization titer is associated with the frequency of spike-specific MBCs after the first vaccine dose (A) Spike-specific MBCs (CD19^+^ CD20^+^ CD38^lo/mid^ IgD-excluding switched naive CD27^−^ CD21^+^ cell) according to dual positivity for spike-PE and spike-APC to exclude non-specific binding in a representative naive pre-vaccine sample (left) or representative post-vaccine sample (right) after the first vaccine dose. (B) Percentage of spike-specific MBC after the first vaccine dose stratified by prior SARS-CoV-2 infection. Line represents median for each group. Statistical test: M-Whitney U test (MWU). Dotted lines represent lower limit of sensitivity of the assay (0.1% spike-specific MBCs, based on previous optimization).^42^ (C) Percentage of spike-specific MBCs in SARS-CoV-2 naive donors after the first vaccine dose, stratified by delayed (magenta) or standard (gray) neutralization profile. Line represents median for each group. Statistical test: MWU. Dotted lines represent lower limit of sensitivity of the assay (0.1% spike-specific MBCs). (D) Correlation of the percent of spike-specific MBC with WT ID_50_ titers stratified by PLWH (blue) and controls (gray) after the first dose, statistical test: Spearman’s rank correlation coefficient. (E) Distribution of MBCs (CD19^+^ CD20^+^ CD38^lo/mid^ IgD-) subtypes according to CD27-BUV395 and CD21-BV711 in a representative HIV-negative donor sample (left) or PLWH donor sample (right). (F) Percentage of MBC subtypes (activated CD27^+^ CD21^−^; resting CD27^+^ CD21^+^; switched naive; switched naive CD27^−^ CD21^+^ and CD27^−^ CD21^−^atypical) after the first vaccine dose stratified by delayed or standard neutralization profile. Line represents median for each group. Statistical test: MWU. (G) Correlation of the percentage of resting CD27^+^ CD21^+^ MBCs with WT ID_50_ titers stratified by delayed (magenta) or standard (gray) neutralization profile after the first vaccine dose, statistical test: Spearman’s rank correlation coefficient. (H) Correlation of the percent of switched naive CD27^−^ CD21^+^ MBCs with WT ID_50_ titers stratified by delayed (magenta) or standard (gray) neutralization profile after the first vaccine dose, statistical test: Spearman’s rank correlation coefficient. ^∗^p > 0.05; ^∗∗^p > 0.01; ^∗∗∗^p > 0.001 and ^∗∗∗∗^p > 0.0001. See also Figure S2.

Spike is the SARS-CoV-2 glycoprotein and is the sole antigen in most vaccines. It has been previously shown that infection and vaccination produce spike-specific MBCs in proportion to serological responses.^38^^,^^39^^,^^40^^,^^41^^,^^42^ Given that the delay in neutralization observed more frequently in PLWH was not clearly associated with peripheral CD4 T cell counts, we next assessed the relationship with global MBCs and spike-reactive MBC frequency and phenotype, using a previously validated flow cytometry panel, with memory B cells defined as CD19^+^ CD20^+^ CD38^lo/-^ IgD- (Figure S2). This analysis was performed on available PBMC samples after the first vaccine dose, using SARS-CoV-2 naive baseline samples to determine the antigen-specific gate (Figure 2A). We observed a significantly lower frequency of spike-specific MBCs in SARS-CoV-2 naive participants after the first dose as compared to those with a history of prior infection, regardless of HIV status (Figure 2B). Moreover, a lower frequency of spike-specific MBCs was observed in SARS-CoV-2 naive participants who had a delayed neutralization response, although notably there were a small number of donors in the standard neutralization group (Figure 2C). In line with this, the percentage of spike-specific MBCs showed a strong correlation with the nAb titer (Figure 2D) in agreement with previous findings during SARS-CoV-2 convalescence.^42^

Subsequent gating on CD21 and CD27 expression allowed the identification of four populations of class-switched MBCs: CD21^−^ CD27^−^atypical MBCs (also known as tissue-like memory); CD21^−^ CD27^+^ activated MBCs; CD21^+^CD27^+^ classical resting MBCs and CD21^+^ CD27^−^switched naive (also known as intermediate memory) MBCs (Figure 2E) as previously described.^42^ Global defects in the balance of these MBC subsets have been identified previously in PLWH (reviewed in^30^), including those on cART,^43^ with increased numbers of activated and atypical MBCs concurrent with a decrease in resting MBCs. This phenotype is exemplified in (Figure 2E) for a PLWH and an HIV-negative control. We have hypothesized that these inherent defects may have an impact on the quality of serological responses after SARS-CoV-2 vaccination. Global phenotyping of the MBC response after the first vaccine dose revealed that individuals with delayed neutralization, consisting largely of PLWH, had significantly lower numbers of resting MBCs (CD21^+^ CD27^+^) and greater numbers of both CD21^−^ CD27^+^ activated MBCs and CD21^−^ CD27^−^atypical MBCs compared to those with standard neutralization (Figure 2F). Moreover, lower frequencies of resting MBCs correlated with lower nAb titers (Figure 2G), this association is driven by participants with standard neutralization and some individuals with delayed neutralization do have reasonable numbers of resting MBCs. Similarly, higher levels of atypical MBCs significantly correlated with lower nAb titers when considering those with standard neutralization, although the strength of this association was relatively weak (r = −0.4867) (Figure 2H). Frequencies of more than 10% atypical MBCs were only observed in the delayed neutralization group. Together these findings suggest that the MBC subset perturbations seen in PLWH could account for the lower serological output.

### Improved neutralization breadth after the third SARS-CoV-2 dose in PLWH but lower nAb responses persist and are associated with global, but not spike-specific, MBC dysfunction

**Figure 3:**
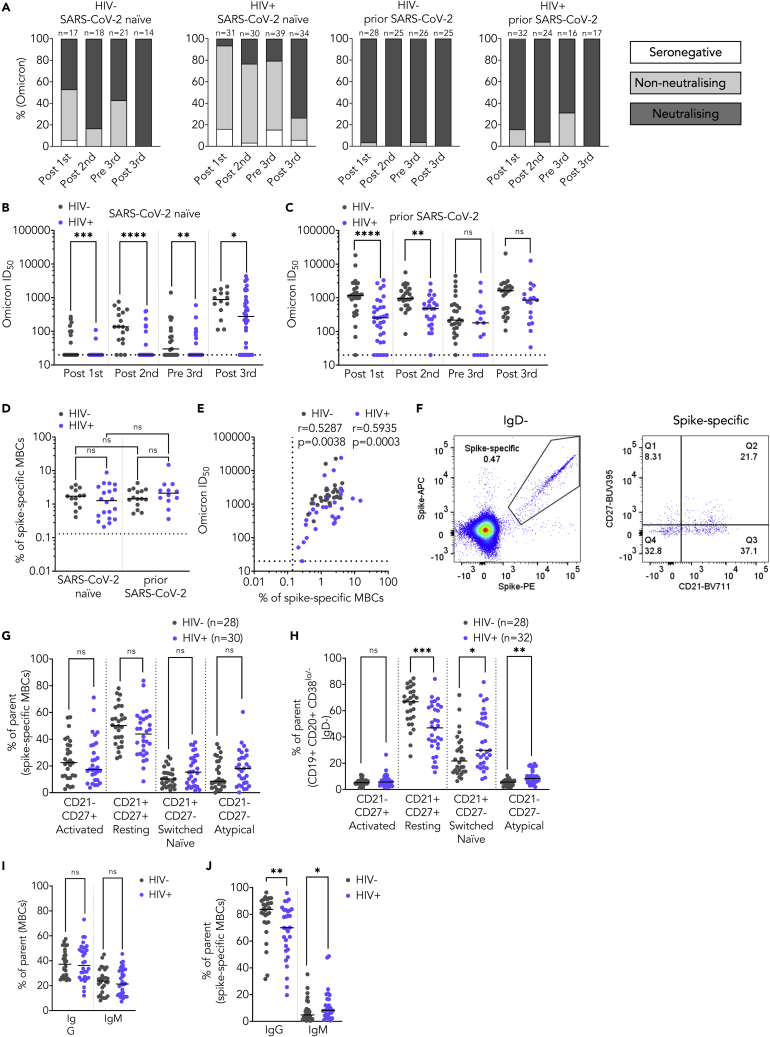
Improved neutralization against Omicron after the third vaccine dose in PLWH accompanied by minimal alteration in the spike-specific MBC phenotype (A) Percentage of individuals with detectable neutralizing response, non-neutralizing but binding response, or seronegative at each timepoint as color-coded in the key (neutralization against Omicron pseudovirus). Headings above each graphshow the HIV status and previous SARS-CoV-2 exposure. N numbers for each group are indicated above each column. (B) Omicron pseudovirus neutralization ID_50_ in PLWH (blue) compared to HIV-negative controls (gray) stratified by vaccination timepoint (on the x axis) for individuals without prior SARS-CoV-2 infection. The dotted line represents the lower limit of the assay (ID_50_ = 1:20). Each data point represents the mean of n = 2 biological repeats, each measured in duplicates. Line represents median for each group. Statistical test: Mann-Whitney U test (MWU). (C) Shows the equivalent data for those with prior SARS-CoV-2 infection, N numbers match those in (A). (D) Percentage of spike-specific MBCs in PLWH (blue) and HIV-negative donors (gray) after the third vaccine dose stratified by SARS-CoV-2 infection. Line represents median for each group. Statistical test: MWU. (E) Correlation between Omicron ID_50_ titers and percentage of spike-specific MBCs in PLWH (blue) and HIV-negative donors (gray) after the third vaccine dose. Statistical test: Spearman’s rank correlation coefficient. (F) Representative gating strategy to identify spike-specific MBCs subtypes. (G) Percentage of spike-specific MBCs subtypes (activated CD27^+^ CD21^−^; resting CD27^+^ CD21^+^; switched naive CD27^−^ CD21^+^ and CD27^−^ CD21^−^atypical) after the third vaccine dose in PLWH (blue) and HIV-negative donors (gray). Line represents median for each group. Statistical test: MWU. (H) Percentage of MBCs subtypes (activated CD27^+^ CD21^−^; resting CD27^+^ CD21^+^; switched naive; switched naive CD27^−^ CD21^+^ and CD27^−^ CD21^−^atypical) after the third vaccine dose in PLWH (blue) and HIV-negative donors (gray). Line represents median for each group. Statistical test: MWU. (I) Percentage of IgG and IgM in MBCs (excluding switched naive CD27^−^ CD21^+^ fraction) after the third vaccine dose in PLWH (blue) and HIV-negative donors (gray). Line represents median for each group. Statistical test: MWU. (J) Percentage of IgG and IgM in spike-specific MBCs after the third vaccine dose in PLWH (blue) and HIV-negative donors (gray). Line represents median for each group. Statistical test: MWU. ^∗^p > 0.05; ^∗∗^p > 0.01; ^∗∗∗^p > 0.001 and ^∗∗∗∗^p > 0.0001. See also Figure S3.

To assess the breadth of nAb responses across the cohort, samples from all timepoints were tested against an Omicron pseudovirus (BA.1 strain), which represented the dominant circulating strain at the time of the post third vaccine dose sampling. Owing to the substantial antigenic changes in the Omicron spike,^44^ in participants with no prior infection, over 50% of HIV-negative controls and more than 90% of PLWH were not able to neutralize Omicron after the first vaccine dose (Figure 3A). The second dose enabled most of the control group to mount a neutralizing response whereas only a quarter of SARS-CoV-2 naive PLWH had nAbs against Omicron. In the SARS-CoV-2 naive groups, the third dose enabled 100% of HIV-negative controls to neutralize Omicron and increased the frequency of neutralization among PLWH to over 70% (Figure 3A). As in the analysis of WT neutralization for individuals without prior SARS-CoV-2 infection, median Omicron ID_50_ titers were lower in SARS-CoV-2 naive PLWH compared to HIV-negative controls at all timepoints (Figure 3B). In addition, there was no significant difference when individuals with complex co-morbidities were removed from the PLWH cohort at the third vaccine dose (Figure S3C) or whether they had previously been infected with SARS-CoV-2. These data suggest that the third vaccine dose was effective in both boosting nAb titer and broadening the response to Omicron, especially in SARS-CoV-2 naive PLWH, thus rendering their responses closer to those of SARS-CoV-2 naive HIV-negative controls (Figures 3B and 3C).

Next, we evaluated cross-sectionally the B cell phenotype after the third vaccine dose. In contrast to the first vaccine dose, there was no significant difference between the frequency of spike-specific MBCs when individuals were stratified by whether they had been previously infected with SARS-CoV-2 or not (Figure 3D) regardless of HIV status. However, the frequency of spike-specific MBCs after the third dose correlated with Omicron titers (Figure 3E). This suggests that after three vaccine doses these individuals had mounted a specific B cell response, and that the quantity of spike-specific B cells remained linked to the improved neutralization potency and breadth observed (Figures 3A–3C). Given that all individuals assessed after the third dose made a robust spike-specific MBC response, we wanted to evaluate further whether alterations in spike-specific MBC phenotype also contributed to differences in serum neutralization (Figures 3A, 3B, 3F, S3A, and S3B). Spike-specific B cells were found to be comparable across the different MBC subsets in both PLWH and HIV-negative controls, except for a trend to fewer spike-specific resting MBCs in PLWH as compared to controls (Figure 3G). This was the case even though the global MBC population for these post third vaccine dose samples showed classical anomalies in MBCs associated with HIV infection (Figure 3H). These data suggest that SARS-CoV-2 serum antibody responses are lower potentially because of a global MBC disturbance thereby limiting the overall B cell response. In line with this proposal, we anticipated that underlying global MBC disturbances would also influence the efficiency of the antigen-specific B cell response in other ways, beyond limiting the number of spike-specific MBCs, for example by limiting class-switching. Indeed, this is supported by our data showing similar levels of IgG+ and IgM+ global MBCs in both groups (Figure 3I) but a significantly lower level of spike-specific IgG+ MBCs in PLWH after the third vaccine dose as compared to controls, and conversely a higher frequency of spike-specific IgM+ MBCs (Figure 3J).

### SARS-CoV-2 vaccination induces robust T cell responses that cross-recognize variants in PLWH

**Figure 4:**
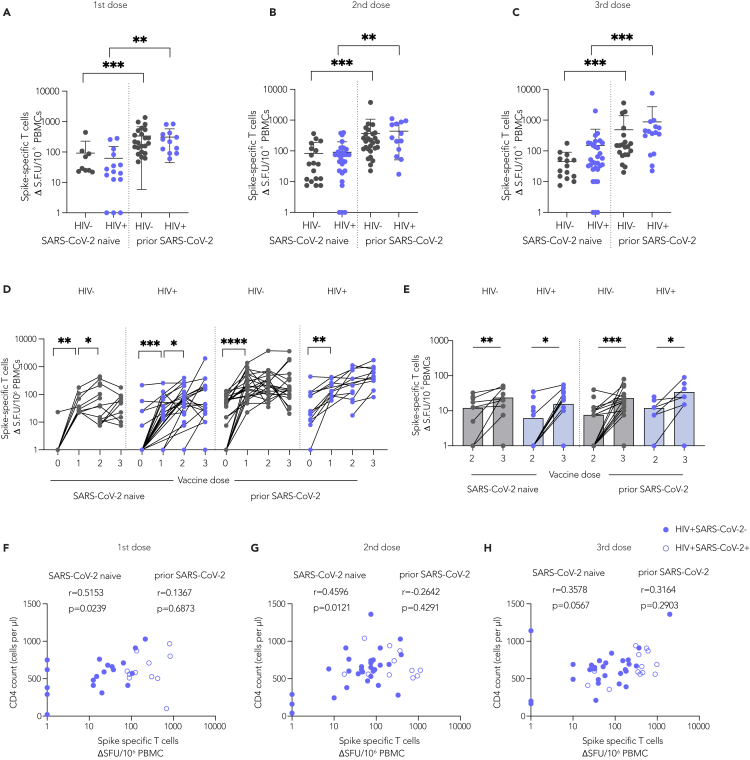
Comparable magnitude of spike-specific T cell responses following SARS-CoV-2 vaccination in HIV-positive and HIV-negative individuals (A–C) Cross-sectional analysis of the magnitude of the IFN-γ-ELISpot responses to the SARS-CoV-2 spike peptide pools in HIV-negative (gray) and HIV-positive (blue) individuals, with or without prior SARS-CoV-2 infection following first dose (A) second dose (B) and third dose (C). (HIV-SARS-CoV-2- first dose n = 9, second dose n = 18, third dose n = 14; HIV+ SARS-CoV-2- first dose n = 15, second dose n = 29, third dose n = 31; HIV-SARS-CoV-2+ first dose n = 23, second dose n = 27, third dose n = 20; HIV+ SARS-CoV-2+ first dose n = 12, second dose n = 13, third dose n = 15). Statistical test: Mann-Whitney U-test (MWU), line represents mean with SD for each group. (D) Longitudinal analysis of the spike specific T cell responses in PLWH and HIV-negative subjects. Statistical test: Wilcoxon matched-pairs sign rank test (WMP). (E) Longitudinal and cross-sectional analysis of the magnitude of T cell responses to Omicron after two or three vaccine doses (n = 11 HIV-SARS-CoV-2-, n = 20 HIV + SARS-CoV-2-, n = 22 HIV-SARS-CoV-2+, n = 10 HIV + SARS-CoV-2+). Statistical test: MWU and WMP. (F–H) Correlation between the CD4 T cell count in HIV-positive individuals and magnitude of spike-specific T cell responses after first dose (F), second dose (G), and (H) third dose. Statistical test: Spearman’s rank correlation coefficient. ^∗^p > 0.05; ^∗∗^p > 0.01; ^∗∗∗^p > 0.001 and ^∗∗∗∗^p > 0.0001. See also Figure S4.

To increase our understanding of the complementary role of cellular immunity after vaccination, we have examined T cell responses in our cohort, including their reactivity to SARS-CoV-2 variants. The magnitude of spike-specific T cell responses was assessed cross-sectionally by IFN-γ-ELISpot using overlapping peptide (OLP) pools covering the complete sequences of the WT spike glycoprotein as previously described.^45^ The majority of PLWH had detectable SARS-CoV-2-specific T cell responses at levels comparable to HIV-negative individuals following each vaccine dose (Figures 4A–4C). A greater magnitude of spike-specific T cells was observed in individuals with prior SARS-CoV-2 infection, irrespective of HIV status (Figures 4A–4C) in keeping with previous reports.^34^^,^^46^^,^^47^ There were no detectable T cell responses in a small number of PLWH with no prior exposure to SARS-CoV-2 across all timepoints. These were participants with incomplete immune reconstitution on cART and/or additional co-morbidities, such as transplant recipients on immunosuppressive therapy (Figures 4A–4C). Next, we examined the longitudinal evolution of T cell responses in a subgroup of donors with available PBMC samples. In SARS-CoV-2 naive individuals, spike-specific T cell responses increased following the first vaccine dose, peaked after the second dose and were maintained after the third vaccine dose (Figure 4D). In one HIV-positive, SARS-CoV-2-naïve donor with advanced immunosuppression and persistently low CD4 T cell count of 100 cells/μL on cART, a third dose (mRNA) vaccine was able to elicit a T cell response despite no evidence of neutralization (Figure 4D). A higher proportion of PLWH without prior SARS-CoV-2 infection had detectable T cell responses at baseline compared to HIV-negative controls, which could represent the presence of cross-reactive responses to other pathogens, probably to related coronaviruses (Figure 4D).^48^^,^^49^^,^^50^^,^^51^^,^^52^ However, due to the small number of participants with detectable T cell responses at baseline, this study was not powered to detect any association between the presence of cross-reactive T cells and magnitude of vaccine-induced T cell responses. In donors with prior SARS-CoV-2 infection, there was a boosting effect to spike-specific T cells following the first vaccine dose in both study groups (Figure 4D). In parallel we have tested T cell responses to CMV-pp65 and HIV-gag peptide stimulation within the same individuals across all timepoints. Overall, PLWH with no prior exposure to SARS-CoV-2 had robust responses to CMV-pp65 stimulation, as expected given their higher CMV seroprevalence compared to HIV negative donors. CMV-specific responses in these individuals were higher compared to SARS-CoV-2 and Gag-specific responses following each vaccine dose (Figures S4A–S4C). Prior SARS-CoV-2 exposure resulted in comparable SARS-CoV-2 and CMV-pp65 T cell responses after the third vaccine dose in PLWH (Figure S4C). No significant differences were detected between SARS-CoV-2 and CMV-specific responses in HIV-negative individuals (Figures S4A–S4C). Overall, these results demonstrate a robust induction of T cell responses to SARS-CoV-2 vaccination in PLWH despite attenuated antibody responses.

Previous work has demonstrated that T cell responses are largely retained against variants of concern (VOCs), including the highly transmissible BA.1 Omicron variant, and therefore may be important when antibody levels wane or new variants emerge that can partly escape antibody responses. To determine T cell reactivity to VOCs, we assessed T cell responses to the mutated regions, including Omicron, in our study cohort. The magnitude of T cell responses against B.1.1.529 was comparable between PLWH and HIV-negative donors regardless of prior SARS-CoV-2 infection (Figure 4E). Notably, responses were further enhanced by a third vaccine dose in all donors, irrespective of prior SARS-CoV-2 infection or HIV status and in keeping with the beneficial effect of a third vaccine dose in boosting humoral responses (Figure 4E). T cell reactivity to Omicron and other VOCs, including Alpha, Beta and Delta, was comparable between HIV-negative and PLWH with or without prior SARS-CoV-2 infection after three vaccine doses, and these responses were maintained against the ancestral Wuhan Hu-1 spike peptide pool, reinforcing the relative resilience of T cell responses to spike variation (Figures S4D and S4E). We noted that three HIV-negative and five HIV-positive individuals, regardless of prior SARS-CoV-2 infection, had no detectable T cell responses to the Wuhan Hu-1 peptide pool, covering only the affected regions of spike. This could be because of the VOC mutations occurring in regions that are poorly targeted by T cell responses in some individuals.^34^

Although spike-specific T cell responses were detected at similar frequencies across all groups (Figures 4A–4C), there was variation in the magnitude of responses. To better understand the factors underlying this heterogeneity, we examined the role of various HIV parameters.^45^ We have previously reported an association between the CD4:CD8 T cell ratio and total SARS-CoV-2 responses, especially against the nucleocapsid (N) and membrane (M) protein, in PLWH recovering from COVID-19 disease.^45^ No correlation was observed between the CD4:CD8 T cell ratio and spike-specific T cell responses following vaccination in our cohort (Figures S4F–S4H). However, a positive correlation was detected between the CD4 T cell count and spike-specific T cell responses after the first vaccine dose (r = 0.5153) in SARS-CoV-2 naive PLWH (Figure 4F). This association was weaker after the second vaccine dose (r = 0.4596) and non-significant after the third dose (Figures 4G and 4H). Together these observations suggest that an effective helper T cell response could drive the induction of cellular immunity following vaccination in individuals without prior exposure to SARS-CoV-2. However, the lack of an association between CD4 T cell counts and antibody responses further underlines the relative importance of HIV-associated B cell defects in modulating the induction of effective humoral immunity in addition to potential insufficient T cell priming.

### A proportion of PLWH had low or absent nAbs (ID_50_< 1:150) but detectable T cell responses following vaccination

**Figure 5:**
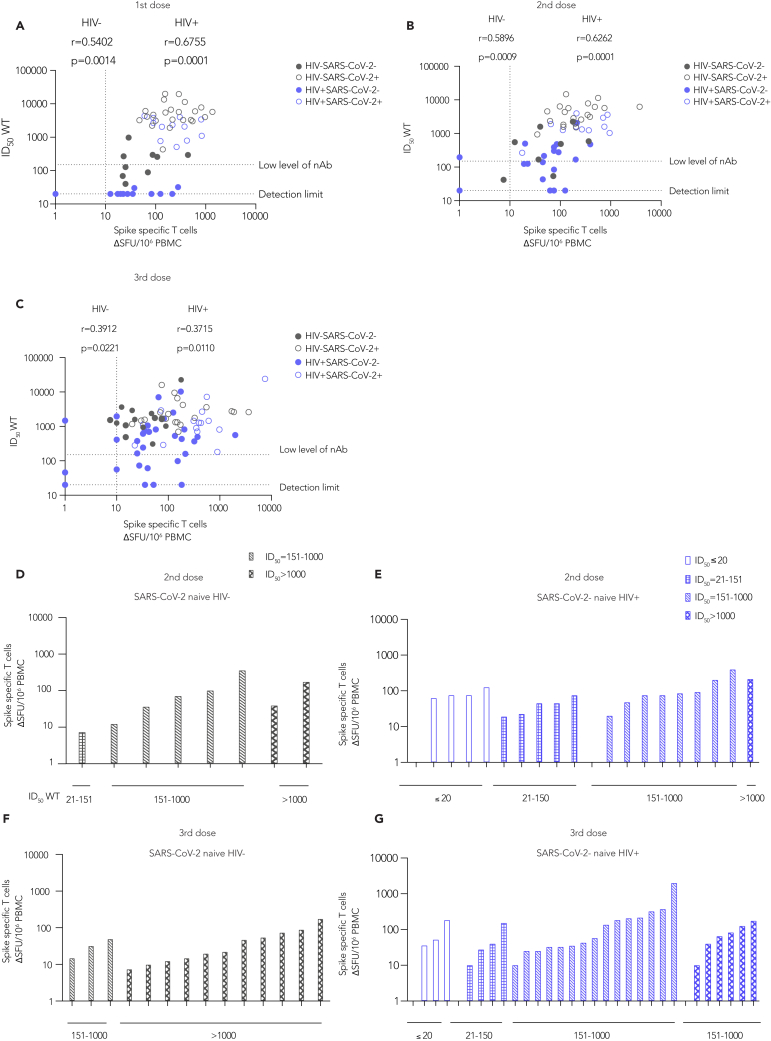
Interrelations between humoral and cellular responses following SARS-CoV-2 vaccination in HIV-positive and HIV-negative individuals (A–C) Correlation of spike-specific T cell responses with nAb titers after first dose (A) second dose (B) and third dose (C) of vaccine in HIV-negative and HIV-positive donors, with or without prior SARS-CoV-2 infection (limit of detection ID_50_ = 1:20, low level of nAb ID_50_ = 1:150). Statistical test: Spearman’s rank correlation coefficient. (D and E) Hierarchy of the spike-specific T cell responses ordered by their nAb titers in HIV-negative (D) and HIV-positive (E) SARS-CoV-2 naive donors after two vaccine doses. (F and G) Hierarchy of the spike-specific T cell responses after three vaccine doses in HIV negative (F) and positive (G) SARS-CoV-2 naive participants. ^∗^p > 0.05; ^∗∗^p > 0.01; ^∗∗∗^p > 0.001 and ^∗∗∗∗^p > 0.0001. See also Figure S5.

We examined next the relationship between humoral and cellular responses by comparing antibody responses and neutralization titers with T cell responses detected by IFN-γ-ELISpot following SARS-CoV-2 vaccination. Overall, spike-specific T cells following the first, second and third vaccine doses correlated positively with respective nAb titers in HIV-negative and PLWH. These associations were stronger in PLWH after the first (r = 0.5402; p = 0.0014) and second dose of vaccine (r = 0.5038, p = 0.0004), similarly to HIV-negative controls (Figures 5A–5C). Similar associations were observed for S1 IgG binding titers (Figures S5A–S5C). One HIV-positive SARS-CoV-2 naive donor with a low CD4 T cell count of 40 cells/μL on cART, and one individual with relapsed lymphoma, both had no detectable humoral and cellular responses after 2 or 3 doses of mRNA vaccine. A proportion of PLWH, in particular those without prior SARS-CoV-2 infection, had low or absent nAbs (ID_50_<1:150) but detectable T cell responses following vaccination (Figures 5A–5C). To better visualize these relationships in SARS-CoV-2 naive individuals, we ranked T cell responses after second and third doses according to the magnitude of neutralizing antibodies (Figures 5E–5G). All of the HIV-negative donors had detectable cellular and neutralizing antibodies (Figure 5D). However, a proportion of SARS-CoV-2 naive PLWH with low or absent nAbs (n = 9 out of 10) had measurable cellular responses to the spike protein after two vaccine doses (Figure 5E). These donors were all controlled on cART with a median CD4 T cell count of 680 cells/μL and no significant underlying co-morbidity (Table S1). Although all HIV-negative individuals had both detectable nAbs and cellular responses post third dose (Figure 2F), a small number of PLWH SARS-CoV-2 naive donors (n = 7 out of 9) had detectable T cell responses in the absence of, or only low-level, neutralization (Figure 5G). Similarly, these donors were all well controlled on cART with a median CD4 T cell count of 492 cells/μL. One of these donors who presented with advanced HIV infection had a persistently low CD4 T cell count (100 cells/μL), and one of the donors recruited after a third vaccine dose had a previous splenectomy. These data suggest that in a small proportion of PLWH, serological non-responders or with evidence of low-level neutralization, cellular immune responses may play an important compensatory role.

### PLWH with suboptimal serological responses demonstrate an expansion of CXCR3^+^CD127^+^ CD8^+^T cells after two doses of SARS-CoV-2 vaccination

**Figure 6:**
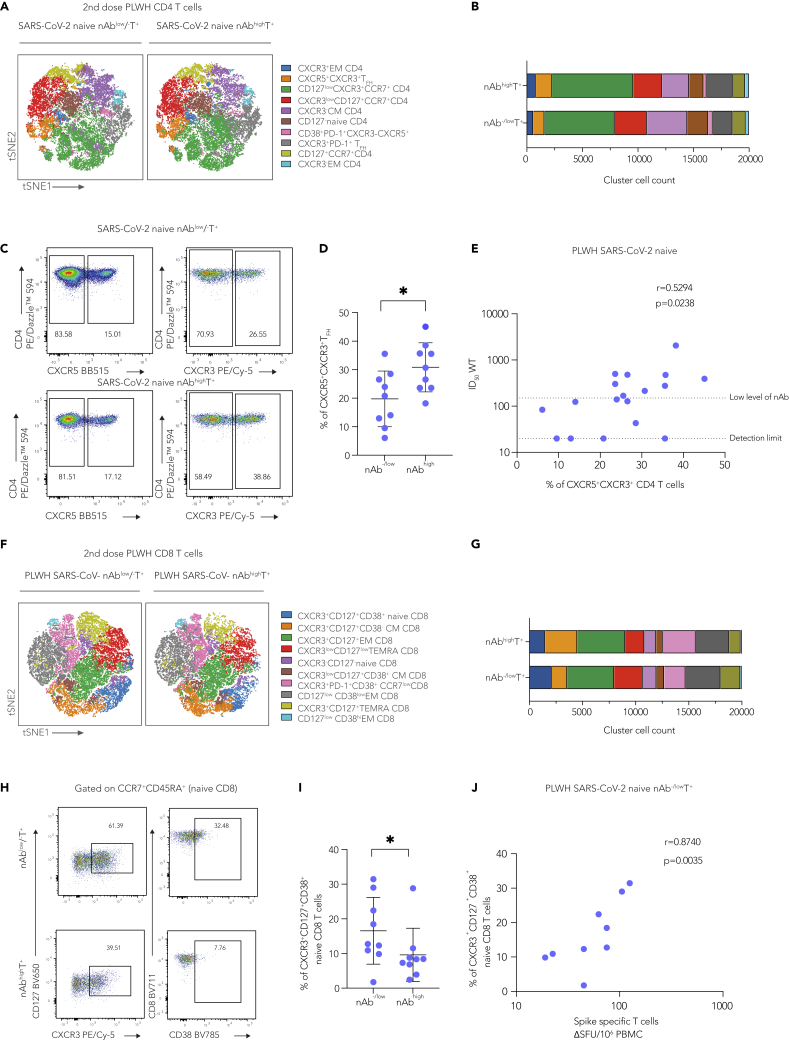
Phenotypic characterization of CD4 and CD8 T cells from SARS-CoV-2 naive HIV positive individuals according to their neutralization levels (A) viSNE map of FlowSOM metaclusters of CD4 T cells from HIV positive SARS-CoV-2 naive subjects after two vaccine doses (nAb^−/low^ = no neutralization or low level of neutralization, nAb^high^ = high neutralization level; n = 9 in each group). Each point on the high-dimensional mapping represents an individual cell, and metaclusters are color-coded. (B) Cell count of each FlowSOM metaclusters out of total CD4 T cells (20,000 cells/group). (C) Representative flow plots from a nAb^−/low^ and nAb^high^ SARS-CoV-2 naive HIV-positive donor showing expression of CXCR5 and CXCR3 within CD4 T cells. (D) Summary analysis of the percentage of CXCR5^+^CXCR3^+^CD4 T cells (n = 9 for each group). Statistical test: Mann-Whitney U-test (MWU), line represents mean with SD for each group. (E) Correlation between frequency of CXCR5^+^CXCR3^+^CD4 T cells and ID_50_ neutralization level in nAb^−/low^ and nAb^high^ SARS-CoV-2 naive HIV-positive individuals after two vaccine doses. Statistical test: Spearman’s rank correlation coefficient. (F) viSNE map of FlowSOM metaclusters of CD8 T cells from nAb^−/low^ and nAb^high^ HIV-positive SARS-CoV-2 naive subjects after two doses of the vaccine (n = 9 in each group). (G) Cell count of each CD8 FlowSOM metaclusters out of total CD8 T cells (20,000 cells/group). (H) Representative flow plots from a nAb^−/low^ and nAb^high^ SARS-CoV-2 naive HIV-positive donor showing expression of CXCR3, CD127, and CD38 within naive CD8 T cells. (I) Summary analysis of the percentage of CD127^+^CXCR3^+^CD38^+^naive CD8 T cells (n = 9 for each group). Statistical test: MWU, line represents mean with SD for each group. (J) Correlation between proportion of CD127^+^CXCR3^+^CD38^+^naive CD8 T cells and SARS-CoV-2 specific T cell responses in nAb^−/low^ HIV-positive SARS-CoV-2 naive subjects. Statistical test: Spearman’s rank correlation coefficient. ^∗^p > 0.05; ^∗∗^p > 0.01; ^∗∗∗^p > 0.001 and ^∗∗∗∗^p > 0.0001. See also Figure S6.

The presence of detectable T cell responses in a subgroup of SARS-CoV-2 naive HIV-positive donors with low or absent nAbs after two or three vaccine doses prompted us to further evaluate the phenotype of the T cell compartment. We have compared T cell immune signatures in SARS-CoV-2 naive PLWH with potent neutralization titers (>1:150) and functional T cell responses (PLWH SARS-CoV-2 naive nAb^high^T^+^, n = 9), with SARS-CoV-2 naive PLWH with low/absent nAbs and a functional T cell responses (PLWH SARS-CoV-2- nAb^−/low^T^+^, n = 9). Both groups were age and sex matched, well controlled on cART and with a similar median CD4 T cell count (Table S1). We have used an unbiased approach and unsupervised high-dimensional analysis, global t-distributed stochastic neighbor embedding (t-SNE), followed by FlowSOM clustering, in circulating T cell populations in the two groups. Ten major CD4 and CD8 T cell subsets were examined using a combination of various activation and differentiation markers, including CD45RA, CCR7, CD127, CD25, CXCR3, CXCR5, PD-1, and CD38 (Figures 6A, S6A, and S6B). There was no difference in the frequencies of the main T cell subsets in the two groups (Figures S6C and S6D). Among CD4 T cells, there was a reduction in circulating CXCR3^+^CXCR5^+^ T follicular helper (T_FH_) subsets observed in HIV-positive nAb^−/low^ compared to nAb^high^ donors (Figures 6A and 6B). The reduced abundance of CXCR3^+^CXCR5^+^ T_FH_ in nAb^−/low^ HIV-positive subjects was further confirmed by manual gating (Figures 6C, 6D, and S3C). CXCR3^+^CXCR5^+^ T_FH_ cells correlated with SARS-CoV-2 neutralization levels in HIV-positive SARS-CoV-2 naive individuals (r = 0.5294 p = 0.02388) (Figure 6E), suggesting that reduced availability of T_FH_ cells could influence the magnitude of vaccine-induced SARS-CoV-2 antibody responses.

We have next examined the CD8 T cell compartment in the two groups. Notably, a prominent cluster delineated by the expression of CXCR3^+^CD127^+^CD38^+^CCR7^+^CD45RA^+^ was significantly enriched in PLWH SARS-CoV-2- nAb^−/low^T^+^ (Figures 6F, 6G, and S6B). The higher abundance of CXCR3^+^CD127^+^CD38^+^CCR7^+^CD45RA^+^ cells in PLWH SARS-CoV-2- nAb^−/low^T^+^ was further confirmed by manual gating (p = 0.04) (Figures 6H, 6I, and S6C). Correlation analysis of these populations showed a positive association between their frequencies and SARS-CoV-2-specific T cell responses following two vaccine doses in PLWH with nAb^−/low^(Figure 6J), supporting the notion that these subsets could contribute to the observed induction of T cell responses in PLWH who lacked or generated low nAb responses. Overall, our analysis of the global T cell profile of individuals with low/absent nAbs but detectable functional T cell responses revealed that reduced availability of T_FH_ cells could contribute to the serological defect observed in conjunction with the previously highlighted imbalance in MBCs. Moreover, we have identified a subset of CD8 T cells that is overrepresented in PLWH with low/absent nAbs and may enable stronger functional T cell responses, supported by recent findings showing that CXCR3^+^ CD8 T cells are polyfunctional and associated with survival in critical SARS-CoV-2 patients, and have been observed in other immunosuppressed groups.^53^^,^^54^

## Discussion

Accumulating evidence suggests that a broad and well-coordinated immune response is required for protection against severe COVID-19 disease. The emergence of VOCs with increased ability to evade nAbs has reinforced the need for a more comprehensive assessment of adaptive immunity after vaccination, especially in more vulnerable groups including some PLWH. Our data indicate that PLWH who are well controlled on cART, elicited poorer humoral responses, in terms of magnitude and neutralizing ability compared to HIV-negative donors following first, second and third doses of SARS-CoV-2 vaccine. This was related to global B cell but not antigen-specific B cell dysfunction. This suggests that the overall disturbance in memory B cell homeostasis during HIV can limit the amount or quality of serum antibody produced indirectly, potentially by decreasing the total number of B cells available to participate in the antigen-specific response. In contrast, the observation that antigen-specific B cells in these individuals are not overtly dysfunctional suggests that lower serum titers are not the result of antigen-specific B cells failing to respond fully, as has been suggested in some chronic diseases.^27^ In contrast, T cell responses were comparable in the two groups and detectable, even in a small group of PLWH with very poor serological responses, suggesting a potentially important non-redundant immunological role for functional T cells. Overall, our data reinforce the beneficial effect of an additional vaccine dose in boosting adaptive immune responses,^20^ especially against circulating VOCs in this patient group.

Weaker humoral responses were observed in PLWH compared to HIV-negative controls after each dose of vaccine when matched by prior SARS-CoV-2 status. Although the third dose largely narrowed the gap between PLWH and controls, and enabled Omicron neutralization, 13% of SARS-CoV-2 naive PLWH still had no nAbs after 3 vaccine doses. This highlights the importance of repeated vaccination in PLWH and suggests additional doses/targeted vaccines could be merited, especially given 28% of SARS-CoV-2 naive PLWH failed to neutralize Omicron after the third vaccine dose. Owing to known defects in germinal center reactions in chronic HIV infection (as reviewed in^29^^,^^30^^,^^55^ recall responses following vaccination in PLWH are likely impaired resulting in lower titers and narrower neutralization breadth. As such, additional vaccinations to stimulate additional affinity maturation and diversification of the response are likely needed to achieve similar outcomes to those seen in HIV-negative individuals. In support of this, previously, it has been shown that PLWH can benefit from an additional vaccine dose and accelerated schedules during hepatitis B immunization.^56^ Moreover, in other immunocompromised groups, repeated vaccination with a third vaccine dose resulted in similarly improved levels of seroconversion and breadth against VOCs.^57^^,^^58^

Previous studies among similar cohorts of PLWH with undetectable HIV viral loads have produced mixed results, as previously reviewed.^13^ SARS-CoV-2 viral vector vaccines have shown similar magnitude and durability of antibody responses to HIV-negative controls^23^^,^^59^ but reduced levels of seroconversion and neutralization have been reported after two doses in PLWH in a more recent study.^15^ Furthermore, viral vector vaccines, lower CD4 T cell count/viremia and old age have been linked to lower serological responses and breakthrough infection.^16^ In terms of mRNA vaccines, both non-significant^60^^,^^61^ and significant decreases in humoral responses have been reported in PLWH.^62^^,^^63^^,^^64^^,^^65^ These differences may be because of the size of cohorts examined and the range of immune reconstitution in these PLWH. In contrast to previous work,^8^^,^^9^^,^^10^^,^^12^ we have found no association between the CD4 T cell count and serological outcome, which could be because of insufficient power in this study to detect differences. The recent study by^20^ demonstrated a stronger humoral response after the third dose of vaccine in PLWH, regardless of their CD4 T cell count, which is consistent with our findings,^20^ Thus, the lower level of nAbs observed here in PLWH could be in part owing to potential differences in boosting of memory responses to enable breadth against Omicron after three vaccine doses.

Serological data correlated significantly with frequency of spike-specific MBCs. The B cell phenotyping confirmed the characteristic and persistent defects seen in global MBCs in the setting of HIV (reviewed in^30^). Specifically, we have observed lower frequencies of resting MBCs and higher frequencies of atypical and activated MBCs. This dysregulated MBC phenotype was also associated with a delay in developing nAbs after the first dose regardless of HIV status. Further evaluation in a group of individuals after the third vaccine dose led to the interesting observation that although the global MBC landscape is still disrupted with lower levels of resting MBCs and higher levels of atypical and switched naive MBCs in PLWH, this is not reflected in the antigen-specific MBCs. Spike-specific MBCs present in PLWH had a similar memory B cell phenotype as HIV-negative controls, albeit fewer resting MBCs. However, higher levels of global atypical MBCs, also observed in PLWH with lower neutralization at the third vaccine dose, suggest that the excess atypical MBCs may be effectively exhausted, as has been described.^66^ Therefore, SARS-CoV-2 serum antibody responses may be lower not because spike-specific responses are enriched within atypical MBCs and therefore unable to progress to an antibody secreting phenotype (as has been postulated for HIV/HBV^27^^,^^67^), but rather because of global MBC disturbance. Thus, we propose that this reduced nAb to vaccination in PLWH may not be because of an alteration in the phenotype of antigen-specific cells but rather limited numbers of MBCs available to participate in the antigen-specific response via the canonical pathway.

In contrast to serological responses, SARS-CoV-2 vaccination elicited comparable T cell responses between PLWH and HIV-negative controls at all sampling points, and these responses were largely preserved against circulating VOCs, including Omicron, following three vaccine doses. These findings are in line with the recent observations showing a robust T cell response to SARS-CoV-2 after third dose in PLWH, including to known VOCs, with either homologous or heterologous combinations of SARS-CoV-2 vaccines.^19^^,^^20^ Of interest, in a recent study despite the detection of significant T cell responses post a third dose mRNA vaccine in PLWH who had completed an mRNA primary course, these responses were reported to be impaired compared to the general population.^20^ These results contrast our findings and could be attributed to the different study design/vaccine platforms and quantification of T cell responses using whole blood assay stimulation and IFN-γ quantification via ELISA.^20^ Similarly, to the scenario seen in antibody responses, prior SARS-CoV-2 infection also resulted in higher T cell responses to vaccination.^34^^,^^46^^,^^47^ Of interest, detectable T cell responses were noted in a proportion of SARS-CoV-2 naive individuals at baseline,^23^^,^^45^ which could represent pre-existing cross-reactive T cell cells due to past infection with other coronaviruses.^68^ An association between CD4 T cell counts and the magnitude of T cell responses was observed in SARS-CoV-2 naive PLWH following vaccination, highlighting the relevance of immune cell reconstitution in producing effective immunity to vaccination, especially in people who lack memory responses elicited by natural infection. In this cohort, PLWH were well-controlled on cART and had undetectable HIV viral loads. Both PLWH with, and without, prior SARS-CoV-2 exposure had similar median CD4 T cell counts (602 and 560 cells/μL, respectively) despite different serological outcomes. However, the full impact of HIV-related immunosuppression, in addition to other factors, including age, sex and presence of co-morbidities, in dampening effective and long-lived memory responses needs to be addressed in future larger prospective studies. It is possible that different vaccine schedules, i.e., homologous versus heterologous vaccination, could also account for the observed heterogeneity in cellular immune responses. A heterologous viral vectored/mRNA vaccination has been described to lead to increased reactogenicity, combining the advantages from both vaccine classes.^69^ Owing to limited numbers, it has not been possible to address the impact of different vaccine platforms in our cohort. However, previous work has shown that the adenovirus-based platforms induce a higher T cell response^70^^,^^71^^,^^72^ whereas mRNA vaccine generates a stronger antibody response.^72^^,^^73^^,^^74^ The use of heterologous boosting strategies has been shown to expand the quantity and the breadth of T cell immunity and improve the serological response in PLWH.^70^^,^^75^^,^^76^^,^^77^ Therefore, we speculate that different vaccine platforms and/or heterologous versus homogeneous combinations could lead to different magnitude T cell responses in our cohort. However, there are insufficient data to recommend the best vaccine approach to induce a more effective, resilient, and durable response in PLWH. Instead, optimization of vaccine schedules requires a randomized controlled trial to directly compare the immunogenicity of different vaccine platforms to design the most effective vaccination schedules.

Overall humoral responses correlated with the magnitude of T cell responses and our findings corroborate the importance of T_FH_ cells supporting effective B cell responses after vaccination. Notably, in a small subgroup of patients (serological non- or low-level responders), there were detectable T cell responses characterized by a CXCR3+CD127+ CD8 T phenotype. This phenotype was not clearly related to HIV parameters or presence of co-morbidities. These T cell populations have been linked with increased survival in people infected with SARS-CoV-2 and are consistent with observations in patient groups who lack B cell responses.^53^ Upregulation of CXCR3 in vaccine-induced T cells with potential to home to lung mucosa in tuberculosis^78^ suggests that these CD8 T cells described herein could play a role in the protection against severe respiratory diseases such as SARS-CoV-2. One possibility is that in the absence of functional antibody responses, the increased abundance of viral antigens could drive CXCR3+ CD8^+^T cell proliferation, as these cells have been shown to have an enhanced proliferative capacity^79^ and improved effector differentiation.^80^^,^^81^ These CXCR3+ CD8^+^T cells could confer a degree of protection by localization to infected tissue compartments,^79^^,^^82^ and provide site-specific responses, which are known to be important in protection against respiratory disease.^83^ CD8^+^T cells have also been shown to expand following vaccination in patients receiving B cell depleting therapies^84^ and to contribute to vaccine mediated protection against SARS-CoV-2 in rhesus macaques.^85^ However, studies in larger cohorts with breakthrough infections are necessary to clearly evaluate the contribution of these CD8^+^T cell populations in vaccine-mediated protection.

Overall, our data support the benefit of a third SARS-CoV-2 dose in inducing nAbs against Omicron in PLWH, as it does in the general population. Moreover, our study provides new insights into the reasons why some PLWH fail to produce effective humoral responses via an in-depth assessment of B cell responses. Specifically, we find that global B cell dysfunction is related to lower serological output in terms of both binding and neutralizing responses. Antibody responses take longer to develop in individuals with greater global B cell dysfunction, which is most commonly seen in PLWH. Although a third SARS-CoV-2 vaccine dose improves neutralization potency and breadth for many, lower titers and MBC disturbance are still observed. Prospective longitudinal studies are now needed to assess whether global B cell disturbance fluctuates in PLWH on cART over time, what treatments/co-morbidities influence this and what level of B cell dysfunction results in inferior clinical outcomes long-term with regards to infectious diseases, particularly where vaccination has taken place. In parallel, CD8^+^T cell profiles and anti-viral T cell activity should be monitored in such studies to understand whether these cells do provide the proposed immunological compensation for defects in humoral immunity.

### Limitations of the study

Our study has several limitations. These include a cross-sectional analysis, which precludes the establishment of causal relationships. Our cohort is heterogeneous, with differences in sex, age and levels of immunosuppression that may contribute to the variability in the magnitude of responses. Moreover, the current analysis provides an overview of responses after up to three vaccine doses, and therefore further work is required to assess the durability and resilience of these responses against subvariants and additional vaccine doses. In addition, we could not assess the impact of breakthrough infections on humoral and cellular immune response, although some individuals were infected with SARS-CoV-2 after vaccination as noted above, because the numbers of re-infections were too low across any given timepoint for meaningful analysis.

## STAR★Methods

### Key resources table

**Table:** 

REAGENT or RESOURCE	SOURCE	IDENTIFIER
**Antibodies**		

Goat anti-human F(ab)’2	Jackson ImmunoResearch	Cat# 109-006-006; RRID: AB_2337553
Alkaline phosphatase-conjugated goat anti-human IgG	Jackson ImmunoResearch	Cat#109-055-098; RRID: AB_2337608
FITC Mouse Anti-Human IgG	BD Biosciences	Cat # 560952; RRID:AB_10563419
BV786 Mouse Anti-Human CD19	BD Biosciences	Cat # 740968; RRID:AB_2740593
BUV395 Mouse Anti-Human CD27	BD Biosciences	Cat # 563815; RRID:AB_2744349
PE-Cy™7 Mouse Anti-Human IgD	BD Biosciences	Cat # 561314; RRID:AB_10642457
APC/Cyanine7 anti-human IgM Antibody	BioLegend	Cat # 314520; RRID:AB_10900422
Alexa Fluor® 700 Mouse Anti-Human CD20	BD Biosciences	Cat # 560631; RRID:AB_1727447
BV711 Mouse Anti-Human CD21	BD Biosciences	Cat # 563163; RRID:AB_2738040
PE-CF594 Mouse Anti-Human CD38	BD Biosciences	Cat # 562288; RRID:AB_11153122
Brilliant Violet 510™ anti-human CD3 Antibody	BioLegend	Cat # 317332; RRID:AB_2561943
Brilliant Violet 510™ anti-human CD14 Antibody	BioLegend	Cat # 301842; RRID:AB_2561946
APC/Cy7 anti-human CD197 (CCR7)	BioLegend	Cat # 353212; RRID:AB_10916390
Brilliant Violet 650™ anti-human CD127 (IL-7Rα) Antibody	BioLegend	Cat # 351325; RRID:AB_11125369
Brilliant Violet 650™ anti-human CD3 Antibody	BioLegend	Cat # 317324; RRID:AB_2563352
Brilliant Violet 711™ anti-human CD27 Antibody	BioLegend	Cat # 302833; RRID:AB_11219201
Brilliant Violet 785™ anti-human CD38 Antibody	BioLegend	Cat # 303530; RRID:AB_2565893
Alexa Fluor® 700 anti-human CD45RA Antibody	BioLegend	Cat # 304120; RRID:AB_493763
Brilliant Violet 421™ anti-human CD279 (PD-1) Antibody	BioLegend	Cat # 329920; RRID:AB_10960742
PE/Dazzle™ 594 anti-human CD4 Antibody	BioLegend	Cat # 300548; RRID:AB_2563566
Brilliant Violet 711™ anti-human CD8a Antibody	BioLegend	Cat # 301044; RRID:AB_2562906
Brilliant Violet 510™ anti-human CD14 Antibody	BioLegend	Cat # 301842; RRID:AB_2561946
Brilliant Violet 510™ anti-human CD19 Antibody	BioLegend	Cat # 302242; RRID:AB_2561668
BB515 Rat Anti-Human CXCR5 (CD185)	BD Biosciences	Cat # 564624; RRID:AB_2738871
BV605 Mouse Anti-Human CD56	BD Biosciences	Cat # 562780; RRID:AB_2728700
PE-Cy7 Mouse Anti-Human CD25	BD Biosciences	Cat # 335824; RRID:AB_2868687
PE-Cy™5 Mouse Anti-Human CD183	BD Biosciences	Cat # 551128; RRID:AB_394061
PerCP-eFluor 710 Anti-Human CD3	eBioscience	Cat # 46-0037-42; RRID:AB_1834395
APC Anti-Human CD19	BioLegend	Cat # 302212; RRID:AB_314242
Brilliant Violet 421™ Streptavidin	BioLegend	Cat # 405226
anti-human IFN-gamma mAb 1-D1K, purified	Mabtech	Cat # 3420-3-1000; RRID:AB_907282
Anti-human IFN-γ mAb (7-B6-1), biotin	Mabtech	Cat # 3420-6-250; RRID:AB_907273
PE-Streptavidin	Agilent	Cat # PJRS25-1
APC-Streptavidin	Agilent	Cat # PJ25S

**Virus strains**		

SARS-CoV-2 (lineage B) isolate SARS-CoV-2/human/Liverpool/REMRQ0001/2020	Ian Goodfellow (University of Cambridge) isolated by Lance Turtle (University of Liverpool) and David Matthews and Andrew Davidson (University of Bristol)	N/A

**Biological samples**		

Donor blood samples	Mortimer Market Centre and Ian Charleson Day Centre, RoyalFree Hospital	N/A

**Chemicals, peptides, and recombinant proteins**		

PepTivator CMV pp65, human	Miltenyi Biotec	Cat # 130-093-438
PepTivator SARS-CoV-2 Prot_S Delta	Miltenyi Biotec	Cat # 130-128-763
PepTivator SARS-CoV-2 Prot_S Com	Miltenyi Biotec	Cat # 130-127-953
PepTivator® SARS-CoV-2 Prot_S Omicron	Miltenyi Biotec	Cat # 130-129-928
Wuhan Hu-1 Alpha	Biochem Shanghai	Customized
Wuhan Hu-1 Beta	Biochem Shanghai	Customized
Alpha Mutation Pool	Biochem Shanghai	Customized
Beta Mutation Pool	Biochem Shanghai	Customized
BD Cytofix/Cytoperm™ Fixation/Permeabilization Solution Kit	BD Biosciences	Cat # 554714; RRID:AB_2869008
Phytohemagglutinin-L (PHA-L)	Sigma-aldrich	Cat # 11-249-738-001
mmPACT® AMEC Red Substrate, Peroxidase	2B Scientific	Cat # SK-4285
Vectastain ELite ABC PK-6100	2B Scientific	Cat # PK-6100
LIVE/DEAD™ Fixable Blue Dead Cell Stain	Invitrogen	Cat #L23105
BD™ CompBeads anti-mouse Ig, κ	BD Biosciences	Cat # 552843; AB_10051478
PEI-Max	Polysciences, Inc	Cat # 23966
Biotin	Sigma-Aldrich	Cat #B4639
Imidazole	Sigma-Aldrich	Cat #I2399
Recombinant biotinylated spike protein	This study and^42^	N/A
Recombinant biotinylated RBD protein	This study and^42^	N/A
SARS-CoV-2 spike S1 re protein	Peter Cherepanov Laboratory (The Francis Crick Institute)^32^	N/A

**Critical commercial assays**		

Bright-Glo Luciferase kit	Promega	Cat #E2650

**Experimental models: Cell lines**		

HEK-293T/17	AmericanType Culture Collection	ATCC CRL-11268
FreeStyle™ 293F	Thermofischer Scientific	Cat #R79007
HeLa-ACE2	James Voss Laboratory (The Scripps Research Institute)^86^	N/A
HEK-293T cells expressing Renilla luciferase (Rluc) and SARS-CoV-2 Papain-like protease-activatable circularly permuted firefly luciferase (FFluc)	Nicholas Matheson Laboratory (University of Cambridge)^87^	N/A

Recombinant DNA		

HIV-1 luciferase reporter vector (pCSLW)	Seow et al.^88^	N/A
HIV-1 packaging construct (p8.91)	Zufferey et al.^89^	
SARS-CoV-2 spike WT (Wuhan hu-1)	Katie Doores Laboratory (King’s College London)^88^	N/A
SARS-CoV-2 spike Omicron (BA.1/B.1.1.259.1)	Katie Doores Laboratory (King’s College London)	N/A
RBD-Avi-His tag	Katie Doores Laboratory (King’s College London)	N/A
SARS-CoV-2 Spike Avi-His tag	Katie Doores Laboratory (King’s College London)^36^	N/A
BirA	Addgene	Cat # 20856

**Software and algorithms**		

Flowjo 10.8.1	FlowJo, LLC	https://www.flowjo.com
GraphPad Prism 9.0.0	GraphPad	https://www.graphpad.com
Cytobank	Beckman Coulter	https://premium.cytobank.org

**Other**		

MSCRN-IP DURA 0.45UM CLEAR 50/PK	Millipore	Cat # MAIPN4550

### Resource availability

#### Lead contact

More information and requests for reagents should be directed to and will be fulfilled by the lead contact, Laura E McCoy (l.mccoy@ucl.ac.uk).

#### Materials availability

This study did not generate unique resources or materials.

### Experimental model and subject details

#### Ethics statement

The protocols and study documents for the study were approved by the local Research Ethics Committee (REC) Berkshire (REC 16/SC/0265) and South Central - Hampshire B (REC 19/SC/0423). All subjects enrolled into the study provided written informed consent. The study complied with all relevant ethical regulations for work with human participants and conformed to the Helsinki declaration principles and Good Clinical Practice (GCP) guidelines.

#### Patient recruitment and sampling

There were 110 HIV + participants who were virally suppressed and on cART and 64 HIV-negative healthy controls were recruited as part of either the Jenner II or the Vaccine in Clinical Infection (VCI) cohorts. PBMCs and plasma (or serum) were collected at the following timepoints: baseline, post-first dose (≥12 days following the first dose), post-second dose (≤70 days following the second dose), pre-third dose (≥70 days following the second dose) to evaluate waning response,^41^^,^^90^ and post-third dose (>7 days following the third dose). Following each dose days cut-off was chosen to allow development of a de-novo (or recall) immune response following vaccination.^88^ Participants received a mix of available SARS-CoV-2 vaccination (Pfizer-BioNTech’s BNT162b2; Moderna’s mRNA-1273 or Astra-Zeneca’s AZD1222) according to Joint Committee on Vaccination and Immunization, UK, guidelines.^4^ Not every participant was sampled at all timepoints. At each visit, participants were asked to report any history of SARS-CoV-2 infection.

Between vaccinations, 7 previously SARS-CoV-2 naïve participants (3 HIV-, 4 HIV+) reported a SARS-CoV-2 infection, as such, any subsequent timepoints were moved into the ‘prior SARS-CoV-2 infection’ group for analysis. Similarly, 4 participants with prior SARS-CoV-2 reported a further infection (3 HIV-, 1 HIV+). All participants were recruited at the Mortimer Market Centre for Sexual Health and HIV Research and the Ian Charleson Day Centre at the RoyalFree Hospital (London, UK) following written informed consent as part of a study approved by the local ethics board committee. Additional information about demographic and sampling can be found in Table S1.

### Method details

#### PBMC isolation

Whole blood was collected in heparin-coated tubes. PBMCs were isolated from whole blood via density-gradient sedimentation. Whole blood was first spun via centrifugation for 5 minat 800g. Plasma was then collected, aliquoted and stored at −80°C for further use. Remaining blood was diluted with RPMI (Gibco, Paisley, UK), layered over an appropriate volume of Ficoll (Cytiva, Uppsala, Sweden) and then spun via centrifugation for 20 minat 800g without brake. The PBMC layer was collected and washed with RPMI to be spun via centrifugation for 10 minat 400g. PBMCs were stained with trypan blue and counted using Automated Cell Counter (BioRad, Hercules, California, USA). PBMCs were then cryopreserved in a cryovial in cell recovery freezing medium containing 10% dimethyl sulfoxide (DMSO) (Honeywell, Seetze, Germany) and 90% heat-inactivated fetal bovine serum (FBS) and stored at −80 °C in a Mr. Frosty freezing container overnight before being transferred into liquid nitrogen for further storage. If present, serum separator tubes were spun at 400g for 5 min to collect serum and then stored at −80°C for further use.

#### Semi-quantitative S1 ELISA

This assay was set up previously by our lab.^45^^,^^91^ In a 96-half-well NUNC Maxisorp^TM^ plate (Nalgene, NUNC International, Hereford, UK), three columns were coated overnight at 4°C with 25 μL of goat anti-human F(ab)′2 (Jackson ImmunoResearch, Ely, UK) (1:1000) in PBS, the other nine columns were coated with 25 μL of SARS-CoV-2 WT S1 protein (a kind gift from Peter Cherepanov (Ng et al., 2020), The Francis Crick Institute) at 3 μg/mL in PBS. The next day, plates were washed with PBS-T (0.05% Tween in PBS) and blocked for 1 hour (h) at room temperature (RT) with assay buffer (5% milk powder PBS-T). Assay buffer was then removed and 25 μL of patient plasma at dilutions from 1:50−1:10,000 in assay buffer added to the S1-coated wells in duplicate. Serial dilutions of known concentrations of IgG were added to the F(ab)′2 IgG-coated wells in triplicate to generate an internal standard curve. After 2 h of incubation at RT, plates were washed with PBS-T and 25 μL alkaline phosphatase (AP)-conjugated goat anti-human IgG (Jackson ImmunoResearch) at a 1:1000 dilution was added to each well and incubated for 1 hat RT. Plates were then washed with PBS-T, and 25 μL of AP substrate (Sigma Aldrich, St Louis, Missouri, USA) added. Optical density (OD) was measured using a Multiskan^TM^ FC (Thermo Fisher-Scientific, Horsham, UK) plate reader at 405 nm and S1-specific IgG titers were interpolated from the IgG standard curve using 4PL regression curve-fitting on GraphPad Prism 9 (GraphPad, San Diego, California, UK).

#### Total IgG ELISA

To measure total IgG levels in plasma, a 96-half-well NUNC Maxisorp™ plate (Nalgene) was entirely coated overnight at 4°C with 25 μL of goat anti-human F(ab)′2 (1:1000). As above, plates were washed in PBS-T and blocked for 1 hat RT in assay buffer. 25 μL of serial dilutions of patient plasma (1:100 to 1:10^7^) were added in duplicates to the plate alongside known concentrations of IgG in triplicates. As above, after 2 h of incubation at RT, plates were washed with PBS-T and 25 μL AP-conjugated goat anti-human IgG was added and then incubated for 1 hat RT. Plates were washed with PBS-T, and 25 μL of AP substrate added. ODs were measured using a Multiskan™ FC plate reader at 405 nm and total IgG titers interpolated from the IgG standard curve using 4 PL regression curve-fitting on GraphPad Prism 9.

#### IgG purification

As the PLWH participants in this study were on cART which can interfere with the lentivirus-based pseudotype neutralization assay IgG was purified from plasma using a Pierce 96-well protein G spin plate (ThermoFischer Scientific). Plasma was incubated in wells containing protein G at RT for 30 min. The captured IgG was then eluted with 0.1M Glycine (pH = 2-3) twice into 2M Tris (pH = 7.5-9) buffer. To remove Tris/Glycine buffer from the purified IgG, the eluate was concentrated (Thermo Scientific Pierce Protein Concentrator PES, 50K MWCO, 0.5 mL) and washed thrice at 10000 rpm for 10 min before quantification by measuring absorbance of 280 nm on a NanoDrop^TM^ (ThermoFischer, Rockford, Illinois, UK). The entire volume of purified IgG was then filtered sterile using a 0.22 μm PDVF hydrophilic membrane FiltrEX™ filter plate (Corning, Corning, NY, USA) and stored at 4°C for further use.

#### Pseudovirus production

In a T75 flask, 3 × 10^6^ HEK-293T cells were seeded in 10 mL of complete DMEM Dulbecco’s Modified Eagle’s Medium (Gibco) supplemented with 10% FBS and 50 μg/mL penicillin-streptomycin. The next day, the following transfection mix was prepared: 1 mL of Opti-MEM^TM^ (Gibco); 90 μL of PEI-max (1 mg/mL); 10 μg of p8.91 HIV-1 gag/pol packaging plasmid^89^; pCSLW HIV-1 luciferase reporter vector plasmid^92^ and 5 μg of either SARS-CoV-2 spike plasmid of interest, specifically WT (Wuhan-hu-1) or Omicron (BA.1/B.1.1.529.1)^88^ as indicated in the results section. The transfection mix was left to incubate for 20 min before being added to the cells and left to incubate at 37°C 5% CO_2_ for 72 h before being collected and filtered through a 0.45 μm filter (Millipore, Cork, Ireland) and either used directly in an assay or stored at −80°C.

#### Pseudovirus neutralization

Neutralization assays were performed in 96-well plates by adding either duplicate serial dilutions of neat plasma in complete Dulbecco’s Modified Eagle medium (Thermo Fisher Scientific-UK) (DMEM) starting at 1:20 dilution for HIV-negative samples or the appropriate amount of purified IgG for HIV + samples to give a starting dilution equivalent to 200 or 400 μg/mL of IgG as based on total IgG. These dilutions were incubated with the appropriate amount of filtered pseudotyped virus for 1 h at 37°C 5% CO_2_ before adding 10000/mL HeLa-ACE2 cells^86^ (kind gift from James Voss, The Scripps Research Institute, USA) in 100 μL per well. After a 72 h incubation at 37°C 5% CO_2_, the supernatant was removed, and cells lysed. Bright-Glo^TM^ luciferase substrate (Promega, Madison, Wisconsin, USA) was added, and relative light unit (RLU) values were read on a Glomax® (Promega) or BioTek Synergy^TM^ H1 (Agilent) plate reader. RLU readouts were used to calculate the reciprocal inhibitory dilution at which 50% of the virus activity is neutralized by plasma (ID_50_) for each sample on GraphPad Prism 9.

#### Live neutralization

The SARS-CoV-2 virus used in this study was the wild-type (lineage B) isolate SARS-CoV-2/human/Liverpool/REMRQ0001/2020, a kind gift from Ian Goodfellow (University of Cambridge, UK), isolated by Lance Turtle (University of Liverpool, UK) and David Matthews and Andrew Davidson (University of Bristol, UK)^93^^,^^94^( Plasma was heat-inactivated at 56°C for 30 mins before use, and neutralizing antibody titers at 50% inhibition (NT_50_) measured as previously described.^87^^,^^95^^,^^96^In brief, luminescent HEK293T-ACE2-30F-PLP2 reporter cells (clone B7) expressing SARS-CoV-2 Papain-like protease-activatable circularly permuted firefly luciferase (FFluc) were seeded in flat-bottomed 96-well plates. The next day, SARS-CoV-2 viral stock (MOI = 0.01) was pre-incubated with a 3-fold dilution series of each sample for 2 hat 37°C, then added to the cells. 16 h post-infection, cells were lysed in Bright-Glo^TM^ Luciferase Buffer (Promega) diluted 1:1 with PBS and 1% NP-40, and FFluc activity measured by luminometry. Experiments were conducted in duplicate. To obtain NT_50_, titration curves were plotted as FFluc vs log (serum dilution), then analyzed by non-linear regression using the Sigmoidal, 4PL, X is log(concentration) function in GraphPad Prism. NT_50_ were quantitated when (1) at least 50% inhibition was observed at the lowest serum dilution tested (1:10, or 1:20 for pre-diluted samples), and (2) a sigmoidal curve with a good fit was generated. Samples with no detectable neutralizing activity were assigned an arbitrary NT_50_ equivalent to the lower limit of quantification.

#### Production of biotinylated protein

To produce biotinylated spike and receptor binding domain (RBD) protein, HEK-293F cells were seeded at 1 × 10^6^ cells/mL in Freestyle™ 293 Expression Medium (Gibco). The next day, a transfection mix was prepared (for 200 mL of cells) of 72 μg of spike-Avi-His tag or RBD-Avi-His tag plasmid and 18 μg of BirA plasmid^36^^,^^88^ into 11 mL of Opti-MEM^TM^, alongside 2 mL of PEI-Max® and 3 mL of 10 mM biotin, and left to incubate at 37°C 5% CO_2_ in a shaking incubator for 7 days before harvesting for purification. The supernatant was purified using an imidazole (Sigma-Aldrich) buffer at a final concentration of 20 mM during binding to the His GraviTrap™ (Cytiva) column and 500 mM imidazole for elution. The eluted protein was then concentrated with a 100KD Amicon® Ultra concentrator (Millipore) and washed with PBS before quantification using a NanoDrop^TM^. Biotinylated protein was then further purified through size exclusion chromatography using an AKTA™ pure system with a Superdex® 200 Increase 10/300 GL column (Sigma-Aldrich) to select for fractions containing trimeric spike or RBD protein.

#### B cell phenotypic flow cytometric analysis

As previously described,^42^ 1 μg of biotinylated spike with either streptavidin-conjugated allophycocyanin (APC) (Agilent, Santa Clara, California, USA) or phycoerythrin (PE) (Agilent) and 0.5 μg of biotinylated RBD with BV421 (BioLegend, San Diego, California, USA) were incubated for 30 minutes in the dark to generate fluorochrome-linked biotinylated tetramers. Previously cryopreserved aliquots of 5 × 10^6^ or 10 × 10^6^ cell aliquots of PBMCs were quickly thawed in PBS, then stained with a panel of phenotyping antibodies and biotinylated tetramers (see Table S2) or phenotyping antibodies only for FMO controls. PBMCs were then washed with PBS and fixed in Cytofix/Cytoperm™ (BD, Franklin Lakes, New Jersey, USA) buffer. Compensation controls were prepared according to manufacturer’s instructions using Anti-Mouse Ig, κ CompBeads™ (BD). Samples were acquired on an LSRFortessa™ (BD) flow cytometer. Data was analyzed (see Figure S2 for gating strategy) on FlowJo v10 (BD). For further analysis of the phenotype of spike-specific MBCs, analysis was limited to samples for which at least 50 cells were acquired in the CD19^+^ CD20^+^ CD38lo/- IgD- MBCs (excluding CD27^−^CD27^+^ cells) spike-PE + spike-APC + gate as previously defined.^42^

#### T cell phenotypic flow cytometric analysis

Purified cryopreserved PBMCs samples were thawed and rested for 2 hours at 37°C in complete RPMI medium (RPMI supplemented with penicillin-streptomycin, L-Glutamine, HEPES, non-essential amino acids, 2-Mercaptoethanol, and 10% FBS) (Gibco).^45^ After 2-hour incubation, cells were washed and plated in a 96-round bottom plate at 0.5-1 × 10^6^ per well and stained for chemokine markers (CXCR3, CCR7 and CXCR5) for 30 minutes at 37 °C. Cells were then washed and stained with surface markers at 4 °C for 20 min with different combinations of antibodies (see Table S3) in the presence of fixable live/dead stain (Invitrogen, Eugene, Oregon, USA). After 20 min of incubation, cells were washed with PBS, and fixed with 4% paraformaldehyde for 15 minat RT. Samples were acquired on a LSRFortessa™ X-20 using FACSDiva™ version 8.0 (BD) and subsequent data analysis was performed using FlowJo v10 (BD). The gating strategies used for flow cytometry experiments are provided in Figures 6 and S6.

#### High-dimensional data analysis

Visualization of high-dimensional single-cell data (viSNE)^97^ and FlowSOM^98^ analyses were performed using the Cytobank platform (https://www.cytobank.org). Concatenated files were used to evaluate overall CD4 and CD8 T cell landscape in different groups. Cells were manually gated for lymphocytes, singlets, CD14^−^CD19^−^live cells, CD3^+^ and CD4^+^ or CD8^+^ and then subjected to viSNE analysis. The viSNE clustering analysis was performed on 8 parameters (CCR7, CD45RA, CD127, PD-1, CD38, CXCR5, CXCR3, CD25). Equal event sampling was selected across all samples. FlowSOM was then performed using the same markers outlined previously for viSNE and with the following parameters: number of clusters 100, number of metaclusters 10; the size of clusters 15 pixels (Cytobank default).

#### *Ex vivo* IFN-γ ELISpot assay

This assay was performed using cryopreserved PBMCs samples. Briefly, 96-well ELISpot plates (S5EJ044I10; Merck Millipore, Darmstadt, Germany) pre-wetted with 30 μL of 70% ethanol for 2 min before washing with 200 μL of sterile PBS. Anti-IFN-γ coating antibody (10 μg/mL in PBS; clone 1-D1K; Mabtech, Nacka Strand, Sweden) was then added and the plates incubated overnight at 4 °C. Prior to addition of cells, ELISpot plates were washed with PBS and blocked with R10 (RPMI supplemented with penicillin-streptomycin, L-glutamine, and 10% FBS) for a minimum of 2 hat 37 °C. PBMCs samples were thawed and rested for 2 hours at 37°C in R10. Cells were then added at 2 × 10^5^ cells/well, in duplicate, and stimulated with overlapping peptide pools at 2 μg/mL for 16–18 hat 37 °C. Unstimulated cells were used as a negative control while PHA (10 μg/mL, Sigma-Aldrich) stimulated cells were used as a positive control. Plates were then washed with 0.05% Tween/PBS (Sigma Aldrich) and incubated for 2 h with an IFN-γ detection antibody (1 μg/mL; clone mAb-7B6-1; Mabtech) followed by 1 h incubation with AP-conjugated streptavidin (1:1000 in PBS, Mabtech). Plates were then washed and visualized using the VECTASTAIN® Elite® ABC-HRP kit according to the manufacturer’s instructions (Mabtech). Antigen-specific T cell responses were quantified by subtracting the number of spots in unstimulated cells from the peptide stimulated cells. An additional threshold of >5 SFU/10^6^ PBMCs was used. Participants who lacked T cell responses to the positive stimuli (PHA) or where antigen-specific responses found to be lower than two standard deviations of negative controls were excluded from the results.

#### Overlapping peptide pools

For the detection of antigen-specific T cell responses, purified cryopreserved PBMCs were stimulated with the following peptide pools: (1) Wild-type SARS-CoV-2 spike; SARS-CoV-2 spike PepTivator® protein pools (Miltenyi Biotec, Gladbach, Germany) were used to test T cell responses against full spike proteome. (2) VOC spike-specific peptide pools; the WuhanHu-1 and variant pools containing peptides from the Wuhan Hu-1, Alpha (B.1.1.7), Beta (B.1.351), Delta (B.1.617.2) and Omicron (BA.1/B.1.1.529.1) sequences (9, 19, 32 and 83 peptides, respectively) were used to define T cell responses to mutated Spike sequences in SARS-CoV-2 variants. Alpha and Beta peptide pools were synthesized by GL Biochem Shanghai Ltd, China and previously used in.^34^ The corresponding controls to Alpha and Beta pools with Wuhan Hu-1amino acid sequences were compared in parallel within the same donor. Delta and Omicron pools were obtained from Miltenyi Biotec. (3) Non-SARS-CoV-2 antigens: Peptide pools of the pp65 protein of human cytomegalovirus (CMV) (Miltenyi Biotec), or HIV-1 Gag peptide pools (NIH AIDS Reagent Repository) were used as positive/negative controls.

### Quantification and statistical analysis

All statistical analysis were carried out in GraphPad Prism 9.0 (GraphPad). All tests were two-tailed. Mann-Whitney U-test (MWU) was used to compare unpaired, non-parametric datawhilst Wilcoxon matched-pairs sign rank test (WMP) was used to compare paired, non-parametric data. Non-parametric Spearman test was used for correlation analysis between two sets of data. Where appropriate, median for groups is shown. Statistical significance in the figures is shown as p value >0.05 (∗); >0.01 (∗∗); >0.001 (∗∗∗) and >0.0001 (∗∗∗∗).

## Data Availability

Any additional information required to reanalyze the data reported in this study is available from the lead contact upon request.
