# Supplementary material for: Attenuated humoral responses in HIV after SARS-CoV-2 vaccination linked to B cell defects and altered immune profiles
Source: iScience. 2022 Dec 24;26(1):105862. doi: 10.1016/j.isci.2022.105862 (PMC9788849; doi:10.1016/j.isci.2022.105862)
Supplement: Document S1. Figures S1–S6 and Tables S1–S3 [file mmc1.pdf]

iScience, Volume *26*

Supplemental information

Attenuated humoral responses in HIV after SARS-CoV-2 vaccination linked to B cell defects and altered immune profiles

Emma Touizer, Aljawharah Alrubayyi, Rosemarie Ford, Noshin Hussain, Pehuén Pereyra Gerber, Hiu-Long Shum, Chloe Rees-Spear, Luke Muir, Ester Gea-Mallorquí, Jakub Kopycinski, Dylan Jankovic, Anna Jeffery-Smith, Christopher L. Pinder, Thomas A. Fox, Ian Williams, Claire Mullender, Irfaan Maan, Laura Waters, Margaret Johnson, Sara Madge, Michael Youle, Tristan J. Barber, Fiona Burns, Sabine Kinloch, Sarah Rowland-Jones, Richard Gilson, Nicholas J. Matheson, Emma Morris, Dimitra Peppa, and Laura E. McCoy

Supplemental Information

- **Figure S1: Lower post vaccination antibody responses in SARS-CoV-2 naïve PLWH. Related to Figure 1**
- **Figure S2: Gating strategy for spike-specific and global MBC. Related to Figure 2**
- **Figure S3: Improved neutralization against Omicron after the 3rd dose in PLWH accompanied by minimal alteration in spike-specific MBC phenotype. Related to Figure 3**
- **Figure S4. Magnitude of T cell responses to Gag, CMV, and VOCs peptide pools and associations with age, days post vaccination and vaccine platform. Related to Figure 4**
- **Figure S5: Correlation between spike-specific T cell response and S1 IgG titers in HIV-positive and -negative individuals. Related to Figure 5**
- **Figure S6. T cell immunophenotyping (or T cell differentiation) in HIV-negative and HIV-positive donors. Related to Figure 6**
- **Table S1: Cohort Demographics and Clinical Characteristics for PLWH SARS-CoV-2 naïve ($nAb^{-/low}T^{+}$ or $nAb^{+}T^{+}$). Related to table 1**
- **Table S2. Reagents used for B cell phenotypic flow cytometric analysis. Related to STAR Methods**
- **Table S3. Reagents used for T cell phenotypic flow cytometric. Related to STAR Methods**

Figure S1: Lower post vaccination antibody responses in SARS-CoV-2 naïve PLWH. Related to Figure 1

- **(A)** SARS-CoV-2 S1 IgG-specific responses (μg/ml) were measured by semiquantitative ELISA in PLWH in PLWH (blue) compared to HIV-negative controls (grey) stratified by vaccination timepoints for individuals without prior SARS-CoV-2 infection. The dotted line represents lower limit of the assay (0.6μg/ml), each data point is representative of n=2 biological repeats. Line represents the median of each group. N numbers match those in Figure 1A, Statistical test: MWU.
- **(B)** Shows the equivalent data for those with prior SARS-CoV-2 infection.
- **(C)** Correlation between WT ID_{50} titres and S1 IgG-specific (μg/ml) titres stratified by PLWH (blue) and controls (grey) at all timepoints, statistical test: Spearman's correlation.
- **(D)** Correlation between WT ID_{50} titres and live-SARS-CoV-2 (WT) NT_{50} titres for PLWH (blue, n=32) and HIV-negative controls (grey, n=14). Dotted lines represent lower limits of both assays (1:20). Live-SARS-CoV-2 NT_{50} represents a single biological repeat.
- **(E)** WT ID_{50} titres in PLWH (blue) compared to HIV-negative controls (grey) stratified by vaccination timepoint for individuals without prior SARS-CoV-2 infection who received mRNA vaccines. Line represents the median of each group. Statistical test: MWU.
- **(F)** Shows the equivalent data for those with prior SARS-CoV-2 infection
- **(G)** WT ID_{50} titres after at the 3rd dose for SARS-CoV-2 naïve PLWH (blue) stratified into either with or without comorbidities (see table for details) compared to HIV-negative controls (grey). Line represents the median of each group. Statistical test: MWU.
- **(H)** Longitudinal semi-quantitative ELISA titres for HIV-negative controls without prior SARS-CoV-2 infection who provided samples after the first and second vaccine dose and were categorised as exhibiting standard neutralizing response (coloured grey), or delayed neutralization if neutralization was only achieved after the second dose (colour-coded in magenta). N numbers for each category are indicated on the graph.
- **(I)** Shows the equivalent data for PLWH without prior SARS-CoV-2 infection
- **(J)** Shows the equivalent data for HIV-negative controls with prior SARS-CoV-2 infection
- **(K)** Shows the equivalent data for PLWH with prior SARS-CoV-2 infection
- **(L)** Correlation between WT ID_{50} titres and S1 IgG-specific (μg/ml) titres stratified by standard (grey) or delayed neutralization (magenta) at all timepoints, statistical test: Spearman's correlation.
- **(M)** Correlation between WT ID_{50} titres and CD4 count or
- **(N)** CD4:CD8 ratio stratified by standard (grey) or delayed neutralization (magenta) at all timepoints, statistical test: Spearman's correlation.

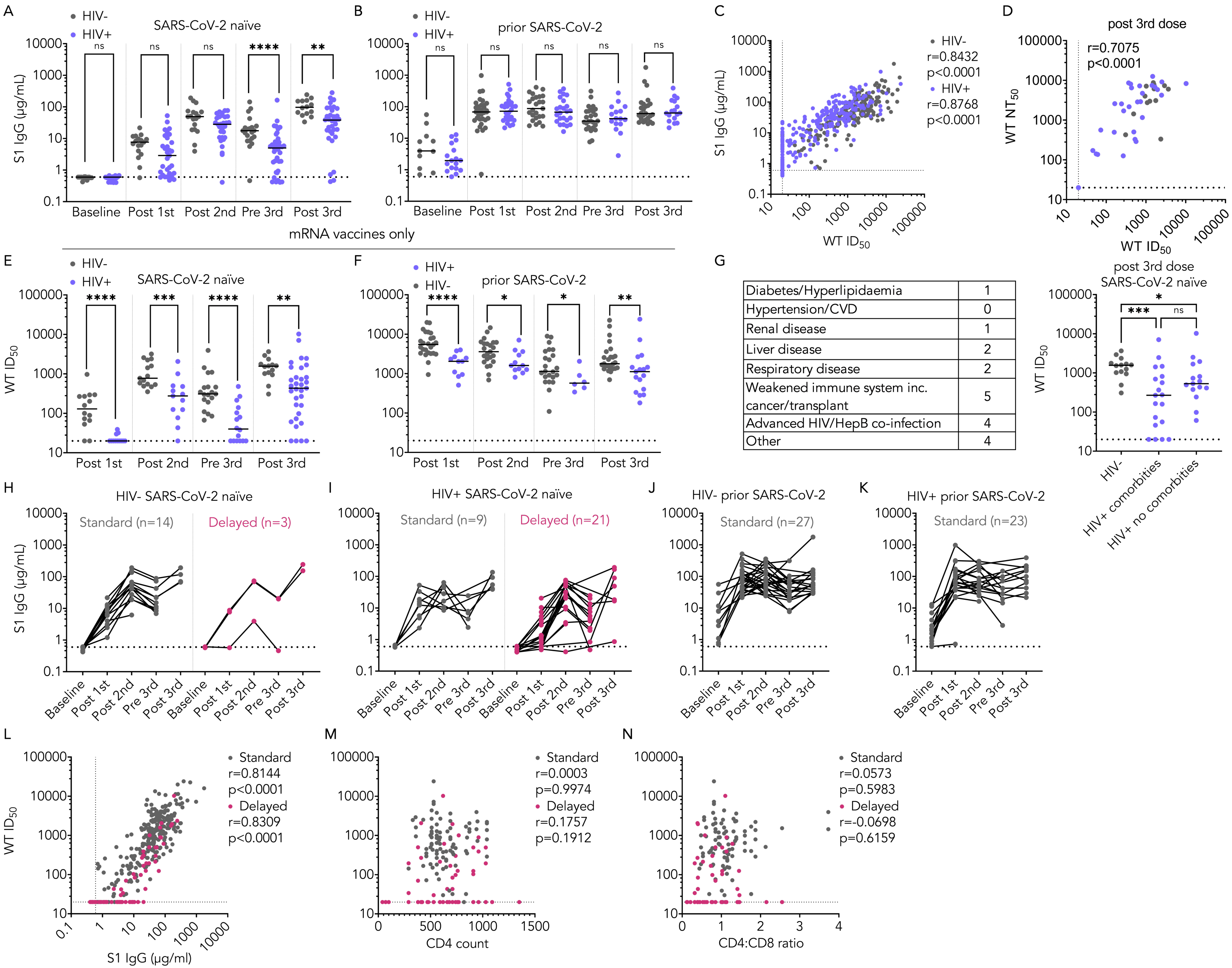
A
HIV-
HIV+
SARS-CoV-2 naïve
S1 IgG (µg/mL)
ns
ns
ns
****
**
Baseline
Post 1st
Post 2nd
Pre 3rd
Post 3rd
B
HIV-
HIV+
prior SARS-CoV-2
ns
ns
ns
ns
ns
mRNA vaccines only
C
S1 IgG (µg/mL)
WT ID50
HIV-
r=0.8432
p<0.0001
HIV+
r=0.8768
p<0.0001
D
post 3rd dose
r=0.7075
p<0.0001
WT NT50
WT ID50
E
HIV-
HIV+
SARS-CoV-2 naïve
WT ID50
****
***
****
**
F
HIV+
HIV-
prior SARS-CoV-2
****
*
*
**
G
Diabetes/Hyperlipidaemia 1
Hypertension/CVD 0
Renal disease 1
Liver disease 2
Respiratory disease 2
Weakened immune system inc. cancer/transplant 5
Advanced HIV/HepB co-infection 4
Other 4
post 3rd dose
SARS-CoV-2 naïve
WT ID50
*
***
ns
HIV-
HIV+ comorbities
HIV+ no comorbities
H
HIV- SARS-CoV-2 naïve
Standard (n=14)
Delayed (n=3)
S1 IgG (µg/mL)
I
HIV+ SARS-CoV-2 naïve
Standard (n=9)
Delayed (n=21)
J
HIV- prior SARS-CoV-2
Standard (n=27)
K
HIV+ prior SARS-CoV-2
Standard (n=23)
L
WT ID50
S1 IgG (µg/ml)
Standard
r=0.8144
p<0.0001
Delayed
r=0.8309
p<0.0001
M
CD4 count
Standard
r=0.0003
p=0.9974
Delayed
r=0.1757
p=0.1912
N
CD4:CD8 ratio
Standard
r=0.0573
p=0.5983
Delayed
r=-0.0698
p=0.6159

Figure S2: Gating strategy for spike-specific and global MBC. Related to Figure 2

Singlet live lymphocytes were first gated on, then using CD3/CD14 to gate out T cells and monocytes and CD19 to gate on B cells. Memory B cells were gated as CD20+ CD38$^{lo/-}$ cells and selecting for class-switched cells (i.e IgD-). This allowed to further gate on specific MBC

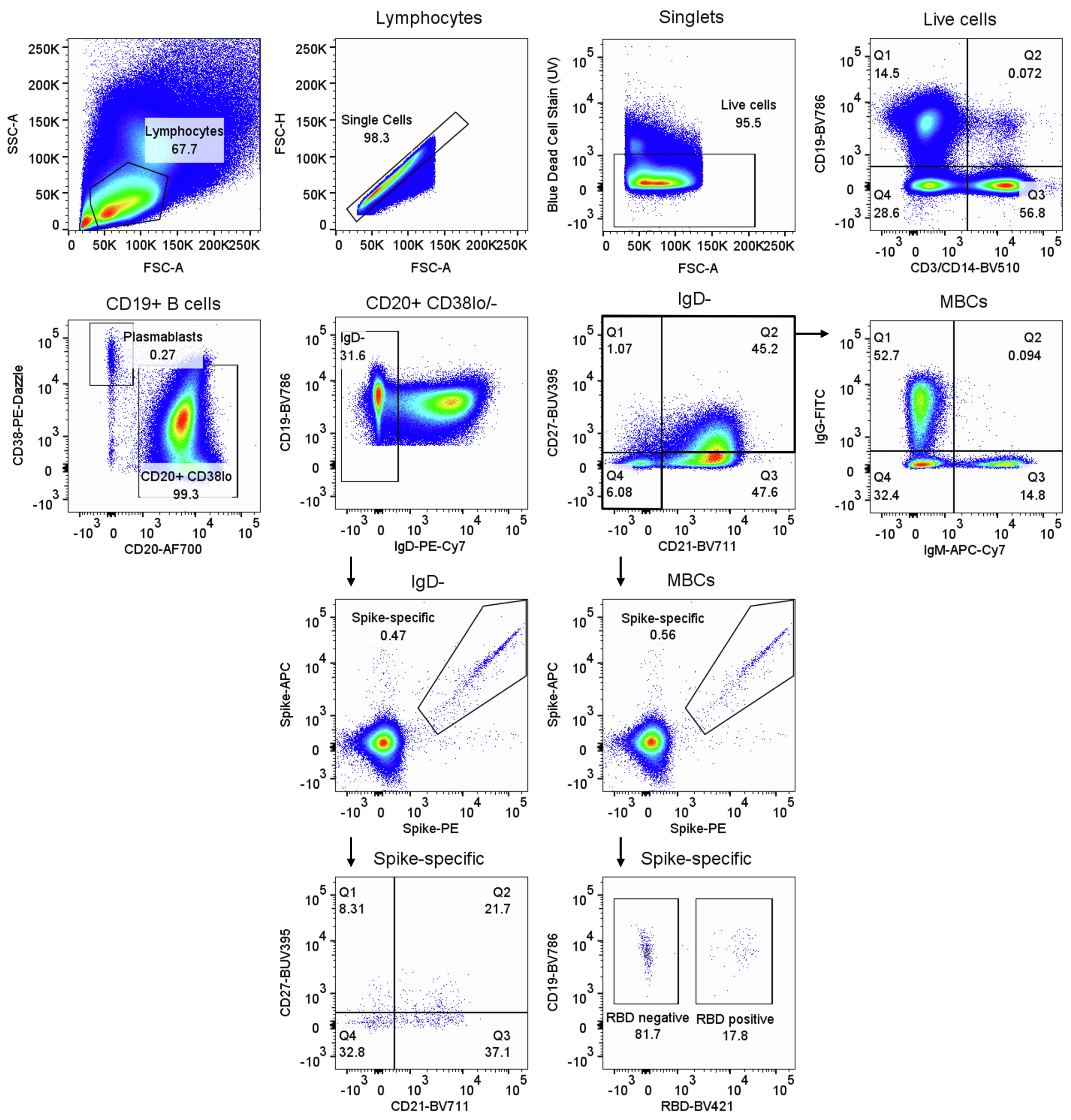

phenotype using CD21 and CD27. 'True' MBCs were defined by excluding the CD21+ CD27- switched naïve population to then gate on isotypes using IgG and IgM or spike-specific MBCs by using biotinylated baits for spike and RBD.

Figure S3: Improved neutralization against Omicron after the 3rd dose in PLWH accompanied by minimal alteration in spike-specific MBC phenotype. Related to Figure 3

- **(A)** WT ID_{50} titres in PLWH (blue) and HIV-negative donors (grey) included in the flow cytometry analysis after the 3rd dose stratified by SARS-CoV-2 infection at the 3rd dose. The dotted line represents the lower limit of the assay (ID_{50}=1:20). Line represents the median of each group. Statistical test: MWU.
- **(B)** Shows the equivalent data as (A) for neutralisation against Omicron
- **(C)** Omicron ID_{50} titres after the 3rd dose for SARS-CoV-2 naïve PLWH (blue) stratified into either with or without comorbidities (see table in Figure 1G for details) compared to HIV-negative controls (grey). The dotted line represents the lower limit of the assay (ID_{50}=1:20). Line represents the median of each group. Statistical test: MWU.
- **(D)** Frequency of spike-specific MBCs after the 3rd dose for SARS-CoV-2 naïve PLWH (blue) stratified into either with or without comorbidities (see table for details) compared to HIV-negative controls (grey). Statistical test: MWU.
- **(E)** Correlation between donor age and WT ID_{50} at post 1st, 2nd, 3rd and pre 3rd dose in SARS-CoV-2 naïve PLWH (blue) and HIV-negative controls (grey). Statistical test: Spearman's correlation.
- **(F)** Correlation between donor age and days since last vaccine dose at post 1st, 2nd, 3rd and post 3rd dose in SARS-CoV-2 naïve PLWH (blue) and HIV-negative controls (grey). Statistical test: Spearman's correlation.

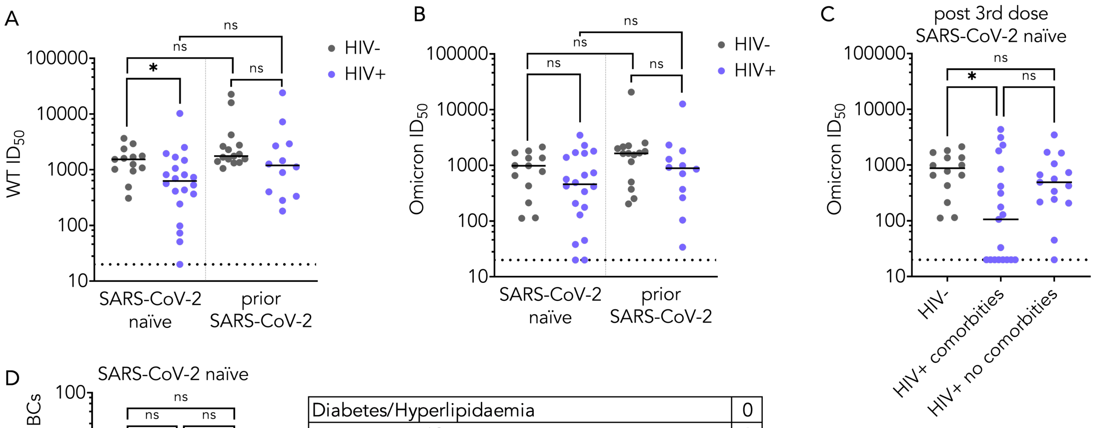

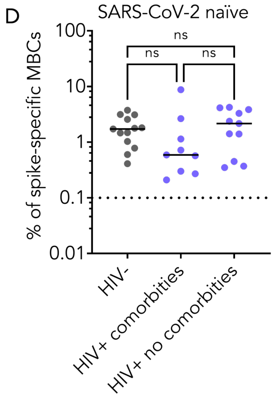

Diabetes/Hyperlipidaemia	0
Hypertension/CVD	0
Renal disease	0
Liver disease	2
Respiratory disease	1
Weakened immune system inc. cancer/transplant	3
Advanced HIV/HepB co-infection	1
Other	2

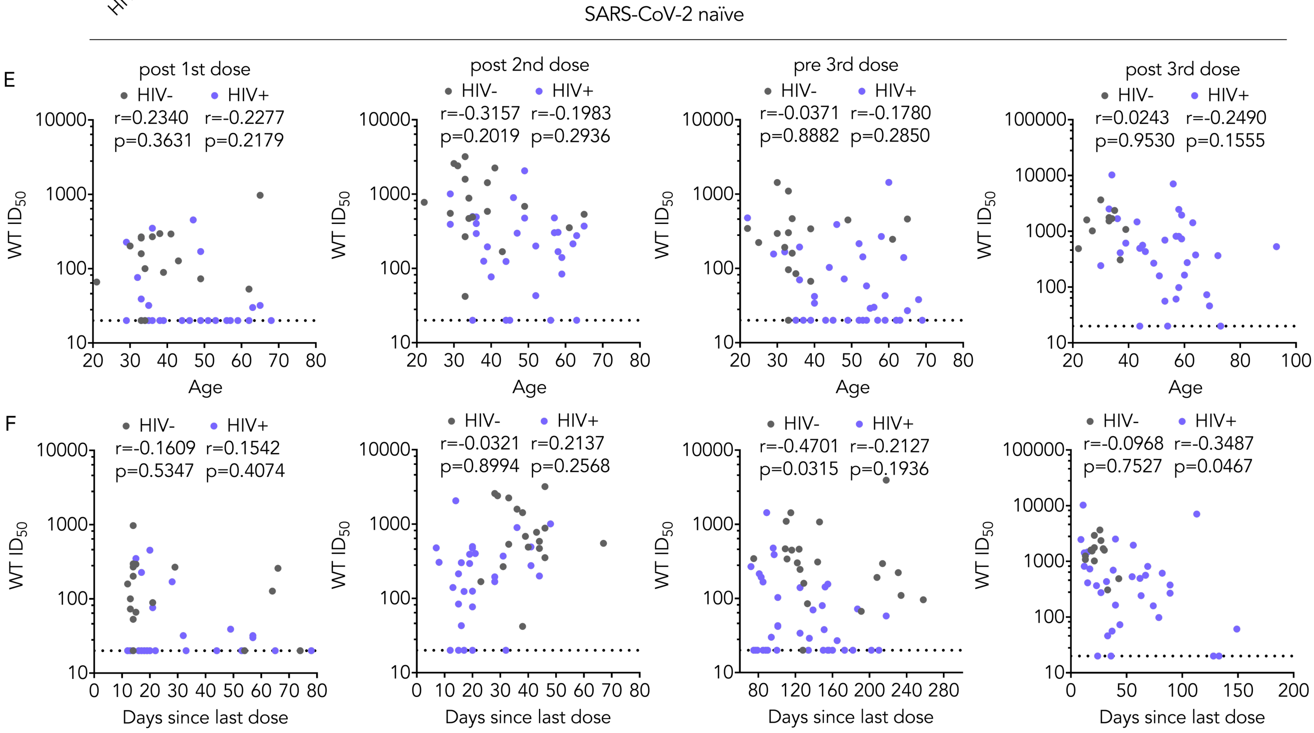

Figure S4. Magnitude of T cell responses to Gag, CMV, and VOCs peptide pools and associations with age, days post vaccination and vaccine platform. Related to Figure 4

(A-C) Paired analysis of the IFN-γ-ELISpot responses for SARS-CoV-2 (spike), CMV (pp65), HIV (Gag), and PHA in HIV-negative and HIV-positive donors after 1st dose (A), 2nd dose (B), and 3rd dose (C) of vaccine. Statistical test: Wilcoxon matched-pairs sign rank test (WMP). Data are represented as mean ± SD.

(D,E) The magnitude of T cell responses to Wuhan, Alpha, Beta, and Delta after three doses of the vaccine in HIV-negative and HIV-positive donors with no prior SARS-CoV-2 infection (D) and with prior SARS-CoV-2 exposure (E). Statistical test: WMP. Data are represented as mean ± SD.

(F-H) Correlation between CD4:CD8 ratio in HIV-infected individuals and the magnitude of spike-specific T cell responses after 1st dose (F), 2nd dose (G), and (H) 3rd dose. Statistical test: Spearman's rank correlation coefficient.

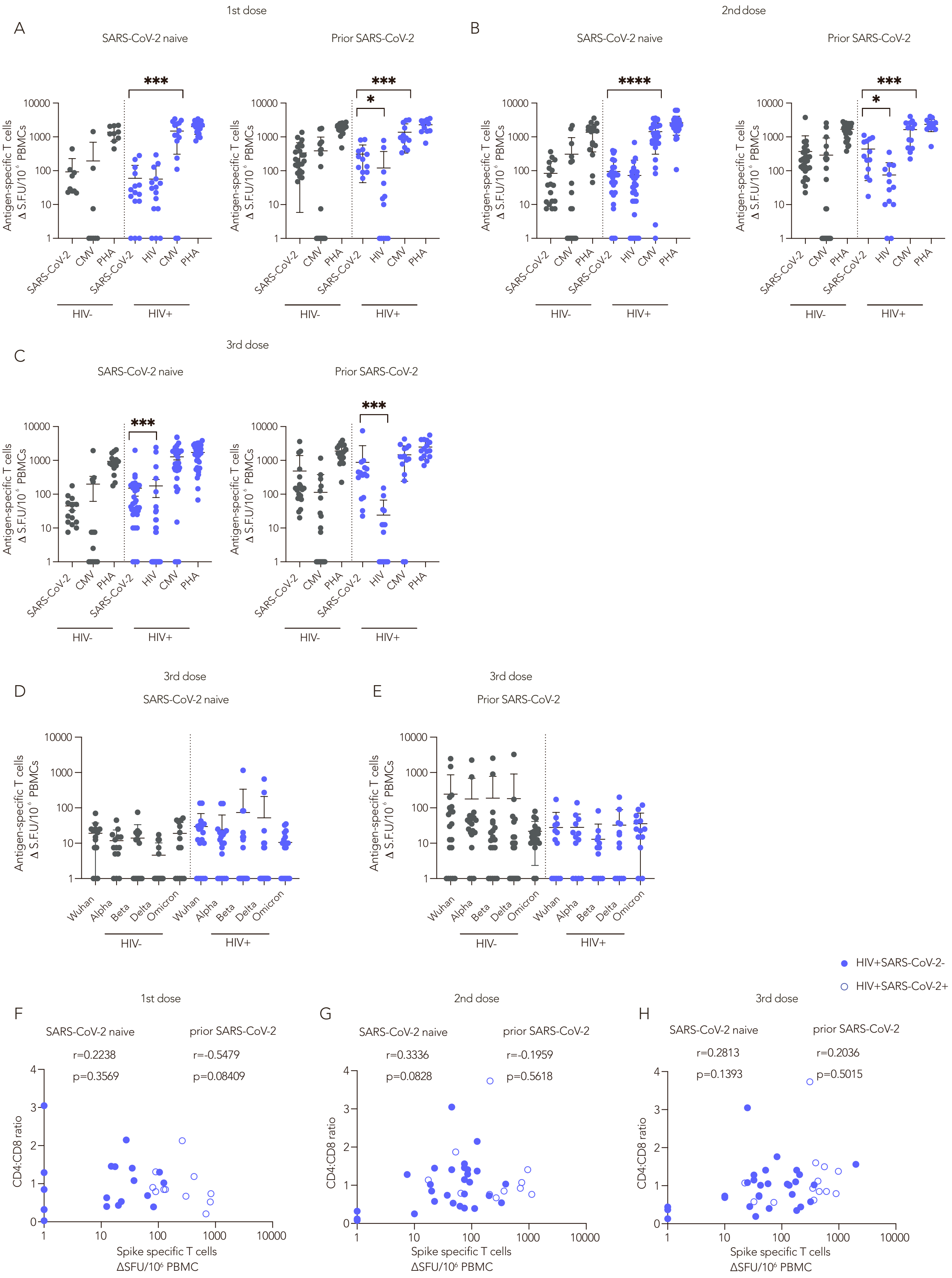
1st dose
A
SARS-CoV-2 naive
Prior SARS-CoV-2
2nd dose
B
SARS-CoV-2 naive
Prior SARS-CoV-2
Antigen-specific T cells
Δ S.F.U/10⁶ PBMCs
SARS-CoV-2
CMV
PHA
HIV
HIV-
HIV+
3rd dose
C
SARS-CoV-2 naive
Prior SARS-CoV-2
3rd dose
D
SARS-CoV-2 naive
3rd dose
E
Prior SARS-CoV-2
Wuhan
Alpha
Beta
Delta
Omicron
HIV+SARS-CoV-2-
HIV+SARS-CoV-2+
1st dose
F
SARS-CoV-2 naive
prior SARS-CoV-2
r=0.2238
p=0.3569
r=-0.5479
p=0.08409
2nd dose
G
SARS-CoV-2 naive
prior SARS-CoV-2
r=0.3336
p=0.0828
r=-0.1959
p=0.5618
3rd dose
H
SARS-CoV-2 naive
prior SARS-CoV-2
r=0.2813
p=0.1393
r=0.2036
p=0.5015
CD4:CD8 ratio
Spike specific T cells
ΔSFU/10⁶ PBMC

Figure S5: Correlation between spike-specific T cell response and S1 IgG titers in HIV-positive and -negative individuals. Related to Figure 5

(A-C) Correlation of spike-specific T cell responses with S1 IgG titers after first dose (A), second dose (B), and third dose (C) of vaccine in HIV-negative and HIV-positive donors, with or without prior SARS-CoV-2 infection (the limit of detection S1 IgG=0.6 µg/ml). Statistical test: Spearman's rank correlation coefficient.

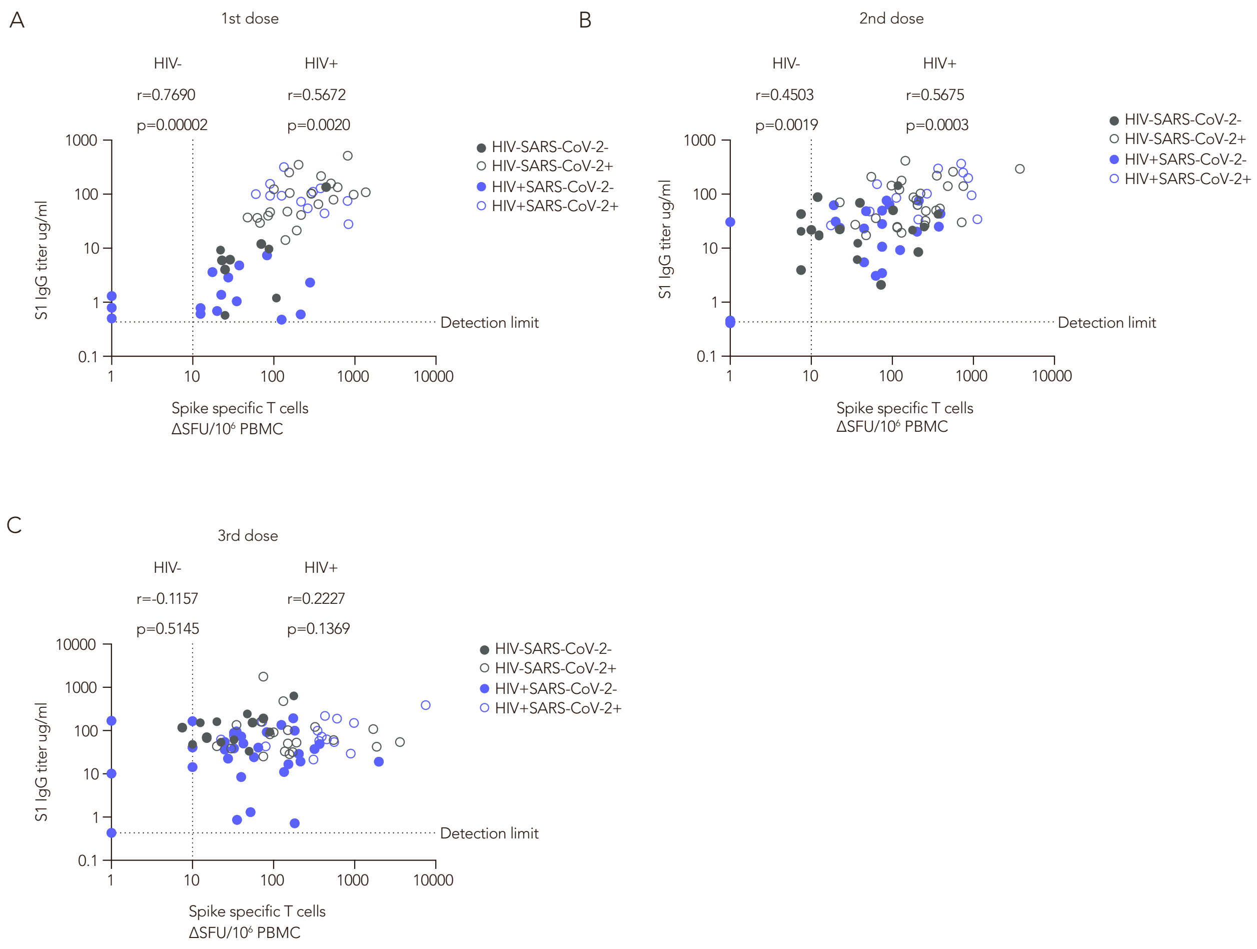

Figure S6. T cell immunophenotyping (or T cell differentiation) in HIV-negative and HIV-positive donors. Related to Figure 6

- **(A)** Surface expression intensity heatmap of the markers indicated for each of the ten FlowSOM meta-clusters of CD4 T cells (showing in Figure. 6 A&B). (colour scale: row z-score expression for each individual marker).
- **(B)** Heatmap of the markers for CD8 T cell clusters (showing in Figure. 3 F&G).
- **(C)** Representative flow plots of the gating strategy for the identification of CM ($CD45RA^{-}/CCR7^{+}$ central memory), naïve ($CD45RA^{+}/CCR7^{+}$), TEMRA ($CD45RA^{+}/CCR7^{-}$ terminally differentiated effector memory) and EM ($CD45RA^{-}/CCR7^{-}$ effector memory) CD4 and CD8 T cells in HIV-negative and HIV-positive donors after two doses of the vaccine.
- **(D)** Summary analysis of the percentage of CD4 and CD8 T cell subsets in $nAb^{-/low}$ (opened blue circle) and nAb^{high} (filled blue circle) individuals (n=9 in each group). Statistical test: Mann-Whitney U-test (MWU). Data are represented as mean ± SD.
- **(E-F)** FlowSOM metaclusters of CD4 (E) and CD8 (F) T cells from $nAb^{-/low}$ and nAb^{high} HIV-positive SARS-CoV-2 naïve subjects after three doses of vaccine (n=5 $nAb^{-/low}$, n=13 nAb^{high}).

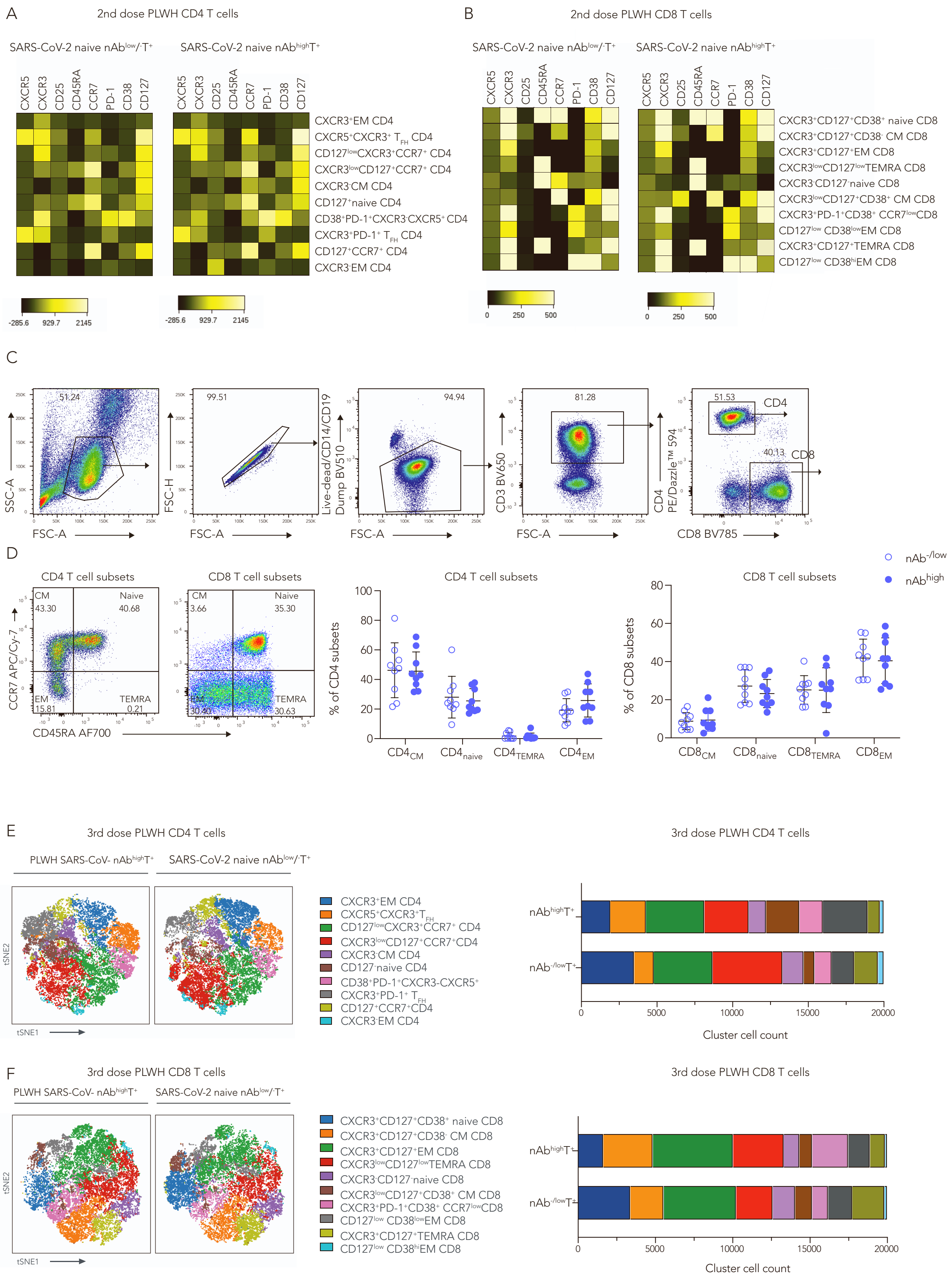
A
2nd dose PLWH CD4 T cells
SARS-CoV-2 naive nAb^low/-T+
SARS-CoV-2 naive nAb^high T+
CXCR5 CXCR3 CD25 CD45RA CCR7 PD-1 CD38 CD127
CXCR3+EM CD4
CXCR5+CXCR3+ TFH CD4
CD127lowCXCR3+CCR7+ CD4
CXCR3lowCD127+CCR7+ CD4
CXCR3-CM CD4
CD127+naive CD4
CD38+PD-1+CXCR3-CXCR5+ CD4
CXCR3+PD-1+ TFH CD4
CD127+CCR7+ CD4
CXCR3-EM CD4
-285.6 929.7 2145
B
2nd dose PLWH CD8 T cells
CXCR3+CD127+CD38+ naive CD8
CXCR3+CD127+CD38- CM CD8
CXCR3+CD127+EM CD8
CXCR3lowCD127lowTEMRA CD8
CXCR3-CD127-naive CD8
CXCR3lowCD127+CD38+ CM CD8
CXCR3+PD-1+CD38+ CCR7lowCD8
CD127low CD38lowEM CD8
CXCR3+CD127+TEMRA CD8
CD127low CD38hiEM CD8
0 250 500
C
SSC-A FSC-A 51.24
FSC-H FSC-A 99.51
Live-dead/CD14/CD19 Dump BV510 FSC-A 94.94
CD3 BV650 FSC-A 81.28
CD4 PE/Dazzle™ 594 CD8 BV785 51.53 CD4 40.13 CD8
D
CD4 T cell subsets
CM 43.30 Naive 40.68 EM 15.81 TEMRA 0.21
CD8 T cell subsets
CM 3.66 Naive 35.30 EM 30.40 TEMRA 30.63
CCR7 APC/Cy-7
CD45RA AF700
% of CD4 subsets
CD4CM CD4naive CD4TEMRA CD4EM
% of CD8 subsets
CD8CM CD8naive CD8TEMRA CD8EM
nAb-/low
nAbhigh
E
3rd dose PLWH CD4 T cells
PLWH SARS-CoV- nAbhighT+
SARS-CoV-2 naive nAblow/-T+
tSNE2 tSNE1
CXCR3+EM CD4
CXCR5+CXCR3+TFH
CD127lowCXCR3+CCR7+ CD4
CXCR3lowCD127+CCR7+CD4
CXCR3-CM CD4
CD127-naive CD4
CD38+PD-1+CXCR3-CXCR5+
CXCR3+PD-1+ TFH
CD127+CCR7+CD4
CXCR3-EM CD4
nAbhighT+
nAb-/lowT+
Cluster cell count
F
3rd dose PLWH CD8 T cells
CXCR3+CD127+CD38+ naive CD8
CXCR3+CD127+CD38- CM CD8
CXCR3+CD127+EM CD8
CXCR3lowCD127lowTEMRA CD8
CXCR3-CD127-naive CD8
CXCR3lowCD127+CD38+ CM CD8
CXCR3+PD-1+CD38+ CCR7lowCD8
CD127low CD38lowEM CD8
CXCR3+CD127+TEMRA CD8
CD127low CD38hiEM CD8
Cluster cell count

Table S1: Cohort Demographics and Clinical Characteristics for PLWH SARS-CoV-2 naïve (nAb$^{-/low}$T^{+} or nAb^{+}T^{+}). Related to table 1

Demographic and clinical characteristics of HIV-positive SARS-CoV-2 naïve individuals who lacked or had low nAbs (ID_{50} <150) but had detectable virus-specific T cell responses following two doses of vaccination.

		HIV+SARS-CoV-2 naïve nAbs$^{low/-}$, T cell responses^{+}	HIV+SARS-CoV-2 naïve nAbs^{high}, T cell responses^{+}
Group size	n	9	9
COVID-19 vaccine	mRNA-based vaccine (BNT162b2/Pfizer or Moderna), n (%)	3 (33.3%)	5 (55.6%)
	ChAdOx1/ AstraZeneca, n (%)	6 (66.7%)	4 (44.4%)
	Day post 2nd dose, median (range)	16 (12-32)	19 (7-41)
Demographics	Age, median (range)	52 (35-63)	57 (29-62)
	Sex, n female:male	1:8	0:9
	Ethnicity, White: BAME	7:2	7:2
HIV parameters	cART	9 (100%)	9 (100%)
	HIV viral load	<50	<50
	CD4, median (range)	680 (470-1360)	650 (380-820)
	CD4:CD8, median (range)	1 (0.4-3.05)	1 (0.39-1.29)
Pre-existing conditions	None, n (%)	7 (77.8%)	6 (66.7%)
	Respiratory disease (asthma and COPD), n (%)	-	1 (11.1%)
	Liver disease, n (%)	1 (11.1%)	-
	Bone disease, n (%)	1 (11.1%)	3 (33.3%)

Table S2. Reagents used for B cell phenotypic flow cytometric analysis. Related to STAR Methods

Antibodies	Supplier	Identifier	Clone	Dilution
FITC Mouse Anti-Human IgG	BD Biosciences	Cat # 560952	Clone # G18-145	5 in 100ul
LIVE/DEAD™ Fixable Blue Dead Cell Stain	Invitrogen	Cat # L23105	-	2 in 1000ul
BV786 Mouse Anti-Human CD19	BD Biosciences	Cat # 740968	Clone # HIB19	2 in 100ul
BUV395 Mouse Anti-Human CD27	BD Biosciences	Cat # 563815	Clone # L128	1 in 100ul
PE-Cy™7 Mouse Anti-Human IgD	BD Biosciences	Cat # 561314	Clone # IA6-2	1 in 100ul
APC/Cyanine7 anti-human IgM Antibody	BioLegend	Cat # 314520	Clone # MHM-88	1 in 100ul
Alexa Fluor® 700 Mouse Anti-Human CD20	BD Biosciences	Cat # 560631	Clone # 2H7	1 in 100ul
BV711 Mouse Anti-Human CD21	BD Biosciences	Cat # 563163	Clone # B-ly4	1 in 100ul
PE-CF594 Mouse Anti-Human CD38	BD Biosciences	Cat # 562288	Clone # HIT2	0.5 in 100ul
Brilliant Violet 510™ anti-human CD3 Antibody	BioLegend	Cat # 317332	Clone # OKT3	0.5 in 100ul
Brilliant Violet 510™ anti-human CD14 Antibody	BioLegend	Cat # 301842	Clone # M5E2	0.5 in 100ul
Brilliant Violet 421™ Streptavidin conjugated to biotinylated RBD (1µl of BV421 to 0.5µg of RBD in 10ul of 1X PBS)	BioLegend	Cat # 405226	-	2 in 100ul
PE-Streptavidin conjugated to biotinylated spike (0.5µl of PE to 1µg of spike in 10ul of 1X PBS)	Agilent	Cat # PJRS25-1	-	2 in 100ul
APC-Streptavidin conjugated to biotinylated spike (1µl of APC to 1µg of spike in 10ul of 1X PBS)	Agilent	Cat # PJ25S	-	2 in 100ul

Table S3. Reagents used for T cell phenotypic flow cytometric. Related to STAR Methods

Antibodies	Supplier	Identifier	Clone	Dilution
APC/Cy7 anti-human CD197 (CCR7)	BioLegend	Cat # 353212	Clone # G043H7	1 in 50ul
Brilliant Violet 650™ anti-human CD127 (IL-7Rα) Antibody	BioLegend	Cat # 351325	Clone # A019D5	1 in 100ul
Brilliant Violet 650™ anti-human CD3 Antibody	BioLegend	Cat # 317324	Clone # OKT3	1 in 100ul
Brilliant Violet 711™ anti-human CD27 Antibody	BioLegend	Cat # 302833	Clone # O323	1 in 100ul
Brilliant Violet 785™ anti-human CD38 Antibody	BioLegend	Cat # 303530	Clone # HIT2	1 in 50ul
Alexa Fluor® 700 anti-human CD45RA Antibody	BioLegend	Cat # 304120	Clone # HI100	1 in 50ul
Brilliant Violet 421™ anti-human CD279 (PD-1) Antibody	BioLegend	Cat # 329920	Clone # EH12.2H7	1 in 100ul
PE/Dazzle™ 594 anti-human CD4 Antibody	BioLegend	Cat # 300548	Clone # RPA-T4	1 in 100ul
Brilliant Violet 711™ anti-human CD8a Antibody	BioLegend	Cat # 301044	Clone # RPA-T8	1 in 100ul
Brilliant Violet 510™ anti-human CD14 Antibody	BioLegend	Cat # 301842	Clone # M5E2	1 in 200ul
Brilliant Violet 510™ anti-human CD19 Antibody	BioLegend	Cat # 302242	Clone # HIB19	1 in 200ul
BB515 Rat Anti-Human CXCR5 (CD185)	BD Biosciences	Cat # 564624	Clone # RF8B2	1 in 50ul
BV605 Mouse Anti-Human CD56	BD Biosciences	Cat # 562780	Clone # NCAM16.2	1 in 50ul
PE-Cy7 Mouse Anti-Human CD25	BD Biosciences	Cat # 335824	Clone # 2A3	1 in 50ul
PE-Cy™5 Mouse Anti-Human CD183	BD Biosciences	Cat # 551128	Clone # 1C6/CXCR3	1 in 25ul
PerCP-eFluor 710 Anti-Human CD3	eBioscience	Cat # 46-0037-42	Clone # OKT3	1 in 100ul
APC Anti-Human CD19	BioLegend	Cat # 302212	Clone # HIB19	1 in 100ul
